# A Continuous Quality Improvement Intervention to Improve Antenatal HIV Care Testing in Rural South Africa: Evaluation of Implementation in a Real-World Setting

**DOI:** 10.34172/ijhpm.2020.178

**Published:** 2020-10-27

**Authors:** H. Manisha Yapa, Wendy Dhlomo-Mphatswe, Mosa Moshabela, Jan-Walter De Neve, Carina Herbst, Awachana Jiamsakul, Kathy Petoumenos, Frank A. Post, Deenan Pillay, Till Bärnighausen, Sally Wyke

**Affiliations:** ^1^The Kirby Institute, University of New South Wales, Sydney, NSW, Australia.; ^2^Africa Health Research Institute, KwaZulu-Natal, South Africa.; ^3^School of Clinical Medicine, Discipline of Obstetrics and Gynaecology, University of KwaZulu-Natal, Durban, South Africa.; ^4^School of Nursing and Public Health, University of KwaZulu-Natal, Durban, South Africa.; ^5^Heidelberg Institute of Global Health (HIGH), Faculty of Medicine and University Hospital, University of Heidelberg, Heidelberg, Germany.; ^6^King’s College Hospital NHS Foundation Trust, London, UK.; ^7^Division of Infection and Immunity, University College London, London, UK.; ^8^Department of Global Health and Population, Harvard T.H. Chan School of Public Health, Boston, MA, USA.; ^9^Institute for Global Health, University College London, London, UK.; ^10^Institute for Health & Wellbeing, University of Glasgow, Glasgow, UK.

**Keywords:** Process Evaluation, Continuous Quality Improvement, Normalisation Process Theory, Tailored Implementation of Chronic Diseases Framew, HIV/AIDS, Antenatal Care

## Abstract

**Background:** We evaluated continuous quality improvement (CQI) targeting antenatal HIV care quality in rural South Africa using a stepped-wedge cluster-randomised controlled trial (Management and Optimisation of Nutrition, Antenatal, Reproductive, Child health, MONARCH) and an embedded process evaluation. Here, we present results of the process evaluation examining determinants of CQI practice and ‘normalisation.’

**Methods:** A team of CQI mentors supported public-sector health workers in seven primary care clinics to (1) identify root causes of poor HIV viral load (VL) monitoring among pregnant women living with HIV and repeat HIV testing among pregnant women not living with HIV, and (2) design and iteratively test their own solutions. We used a mixed methods evaluation with* field notes* from CQI mentors (‘dose’ and ‘reach’ of CQI, causes of poor HIV care testing rates, implemented change ideas); *patient medical records* (HIV care testing by clinic and time step); and *semi-structured interviews* with available health workers. We analysed field notes and semi-structured* interviews* for determinants of CQI implementation and ‘normalisation’ using Normalisation Process Theory (NPT) and Tailored Implementation of Chronic Diseases (TICD) frameworks.

**Results: **All interviewed health workers found the CQI mentors and methodology helpful for quality improvement. Total administered ‘dose’ was higher than planned but ‘reach’ was limited by resource constraints, particularly staffing shortages. Simple workable improvements to identified root causes were implemented, such as a patient tracking notebook and results filing system. VL monitoring improved over time, but not repeat HIV testing. Besides resource constraints, gaps in knowledge of guidelines, lack of leadership, poor clinical documentation, and data quality gaps reduced CQI implementation fidelity and normalisation.

**Conclusion:** While CQI holds promise, we identified several health system challenges. Priorities for policy makers include improving staffing and strategies to improve clinical documentation. Additional support with implementing clinical guidelines and improving routine data quality are needed. Normalising CQI may be challenging without concurrent health system improvements.

## Background

**Key Messages**

** Implications for policy makers**

- Our process evaluation of continuous quality improvement (CQI) in rural South African primary care clinics demonstrates that simple workable solutions were able to address some root causes of low HIV viral load (VL) monitoring among pregnant women living with HIV and repeat HIV testing among pregnant women not living with HIV.
- Despite health worker enthusiasm for improving service quality, resource shortages including staffing shortages, gaps in knowledge of guidelines, poor clinical documentation and data quality were barriers to CQI achieving its full potential in this setting.
- Health system strengthening initiatives in parallel to CQI interventions are essential to optimise HIV care (and other service) quality in resource-limited settings.

** Implications for the public**

Critically examining a programme on improving health service quality is important to identify health system issues that influence the success of the programme. We found that a shortage of vital resources including staffing, knowledge of treatment recommendations, poor clinical documentation and data quality limited effectiveness of a continuous quality improvement (CQI) programme aimed at improving HIV care services for pregnant women in rural South Africa. Our findings contribute to the gaps in knowledge of real-life applications of CQI and similar programmes that might seem desirable for resource-limited settings. Our findings demonstrate the importance of health systems strengthening alongside other initiatives to improve service quality

Continuous quality improvement (CQI) provides a range of time-tested and adaptable techniques to diagnose and manage quality problems in clinics using real-time data. It provides clinical teams with the skills they need to implement evidence-based practice. CQI’s potential to improve quality of health services within available resources makes it an attractive intervention in resource-limited settings^1^ particularly in relation to Sustainable Development Goals.^2,3^ There is a vast literature on “quality improvement” initiatives including audit and feedback,^4,5^ health worker training and supervision,^6-8^ Lean,^9-11^ Six Sigma,^9,10,12,13^ Lean Six Sigma,^10,14^ CQI ([Total Quality Management], the Model for Improvement),^10,15-17^ and others,^18,19^ all aimed at improving service quality. We defined CQI^20^ as that which applies a set of standardised tools used flexibly based on contextual needs, specifically: root-cause analyses performed with process maps^21^ and fishbone diagrams,^22^ then design and testing of local solutions using iterative Plan-Do-Study-Act (PDSA) cycles^23^ with the aid of real-time data trends plotted on run charts.^24^ Although there are some randomised controlled trials (RCTs) underway that test CQI as a *single intervention* in resource-limited primary care settings,^25,26^ to our knowledge there are only 2 completed trials of CQI in such settings.^27-29^ Despite the promise of CQI and rigorous evaluation, these studies failed to demonstrate a positive impact, instead identifying several constraints limiting uptake. Constraints included challenges with staff turnover, lack of available leadership, inadequate ongoing support,^27^ challenges with data quality, staffing shortages, weak infrastructure, and civil unrest.^28,29^

 In settings with high pregnancy and postpartum HIV prevalence and incidence such as South Africa^30-32^ — where prevalence of pre-treatment drug resistance is also increasing^33^ — confirming response to antiretroviral therapy (ART) among women living with HIV, timely revision of the ART regimen among those failing treatment,^34,35^ and early diagnosis of incident HIV for timely ART initiation among women previously not living with HIV,^36^ are essential. Procedural measures of quality on this care pathway are HIV viral load (VL) monitoring of pregnant women living with HIV, and repeat HIV testing of pregnant women not living with HIV. These are critical components of elimination of mother-to-child transmission of HIV (eMTCT) programmes^37,38^ alongside optimising ART adherence^39-41^ and primary prevention of maternal HIV infection.^41-43^ South Africa decentralised HIV care to primary care nurses in order to broaden ART coverage,^44^ and removed CD4+ T-cell eligibility criteria for pregnant and breastfeeding women in January 2015 (Option B+); the guidelines also recommend more frequent HIV VL monitoring and repeat HIV testing for this population.^45^ In September 2016 ART was expanded to all people living with HIV regardless of CD4 count (Universal Test and Treat, UTT).^46^ Amidst a rapidly evolving ART programme and concerns about implementation gaps of these essential HIV care tests,^31,32,34,47,48^ improving antenatal HIV care quality within available resources is crucial.

 We implemented CQI in 7 primary care clinics in rural South Africa between July 2015 and January 2017. We evaluated CQI using a stepped-wedge cluster RCTs, with an embedded process evaluation, to investigate the impact of CQI on quality of antenatal HIV services in rural South Africa.^49^ Our primary endpoints were direct process indicators of antenatal care (ANC) quality designed to eliminate MTCT: (*i*) HIV VL monitoring among pregnant women living with HIV, and (*ii*) repeat HIV testing among pregnant women not living with HIV.^49^ These process indicators, if improved, are expected to have a downstream impact on MTCT. In our primary impact evaluation we found that CQI significantly increased VL monitoring but not repeat HIV testing. Importantly, there were gaps in clinical documentation and HIV care testing rates fell short of expected targets.^50^

 Process evaluations can highlight determinants of intervention uptake and ‘normalisation’ within the broader health system, to inform policy planning prior to scale-up of a seemingly desirable intervention.^51^

 In this paper we present results of our process evaluation of CQI as implemented in our stepped-wedge cluster RCT. We aimed to investigate the process through which the main trial endpoints of VL monitoring and repeat HIV testing were achieved. In particular, we aimed to identify determinants of practice, and whether ‘normalisation’ of CQI into routine services could occur in this setting, by examining the following: (*i*) health worker participation in CQI by describing ‘dose’ and ‘reach’; (*ii*) the ‘black box’ of implemented changes in practice; (*iii*) time trends in endpoint achievements and time to intervention uptake; and (*iv*) CQI mentor and health worker experiences of implementing the intervention.

## Methods

 We first summarise the Management and Optimisation of Nutrition, Antenatal, Reproductive, Child health and HIV care (MONARCH) CQI intervention before describing the process evaluation study design and associated methodology.

###  The MONARCH CQI Intervention

 The MONARCH (NCT02626351) trial has been previously described in detail.^49^ Briefly, the intervention supported nurses and other health workers providing ANC in primary healthcare clinics to identify and implement approaches to improve adherence to 2015 South African national eMTCT guidelines (Table 1), particularly HIV VL monitoring and repeat HIV testing (HIV care tests). Table 1 compares the key changes in VL monitoring and HIV testing guidelines from 2013 to 2015: this helps us interpret the pre-intervention testing rates for each primary endpoint and effort required to integrate new knowledge into routine practice.

**Table 1:** Comparison of 2013 and 2015 South African National eMTCT Guidelines

**Item**	**2013 eMTCTGuidelines** ^a^	**2015 eMTCTGuidelines** ^b^
**ART Criteria**		
	Lifelong ART if CD4+ T-cell count ≤350 cells/mm^3^	Lifelong ART at any CD4+ T-cell count: “Option B+”
	ART prophylaxis during pregnancy and breastfeeding: “Option B” (up to 1 week post cessation of breastfeeding) if CD4 count >350 cells/mm^3^	
**HIV VL Monitoring**		
	At first ANC visit if HIV-positive and on ART	At first ANC visit if HIV-positive and on ART
	At 6 and 12 months post ART initiation	3 and 6 months post ART initiation
		Every 6 months thereafter if VL<1000 copies/mL
		If VL ≥1000 copies/mL repeat within one month with adherence counselling
**HIV Testing**		
	At first ANC visit	At first ANC visit
	3 monthly after first negative HIV test and/or at 32 weeks^c^ or later gestation or during labour	3 monthly during pregnancy
	At 6-week infant immunization visit	During labour/delivery
	At 3, 6, 9 and 12 months during breastfeeding	At 6-week infant immunization visit
		3 monthly during breastfeeding
**Infant HIV Testing**		
	At 6 weeks of age	At birth
	At 6 weeks after cessation of breastfeeding	At 10 weeks of age
	At any other time if clinically indicated	6 weeks after cessation of breastfeeding
	HIV Ab test at 18 months	At any other time if clinically indicated
		HIV Ab test at 18 months

Abbreviations: Ab, antibody; ANC, antenatal care; ART, antiretroviral therapy; eMTCT, elimination of mother-to-child transmission of HIV; VL, viral load. ^a^ National Department of Health South Africa 2013. The South African Antiretroviral Treatment Guidelines: PMTCT Guidelines: 2013. Pretoria. ^b^ National Department of Health South Africa 2015. National Consolidated Guidelines for the Prevention of Mother-to-Child Transmission of HIV (PMTCT) and the Management of HIV in Children, Adolescents and Adults. ^c^Although the 2013 guidelines recommended 3-monthly HIV testing and/or at 32 weeks’ gestation, clinics were re-testing pregnant women at 32 weeks’ gestation prior to receiving the CQI intervention.

 A team of 3 certified CQI mentors (2 isiZulu-speaking nurses and a data clerk) — supported by a data manager, improvement advisor, and scientific advisor (Centre for Rural Health, CRH, University of KwaZulu-Natal) — travelled to the study site from Durban to deliver the intervention.^49^ All CQI mentors had previous experience in supporting CQI in South African healthcare facilities elsewhere. They identified a clinic team of 4-7 health workers at each study clinic (clinic CQI team), guided by the clinic operational manager. They worked collaboratively to guide the clinic CQI team to identify areas for improvement and test solutions. They used standardised CQI tools including fishbone diagrams,^22^ clinical workflow maps (process maps),^21^ PDSA cycles,^23^ and run charts.^24^ Change ideas were implemented flexibly according to clinic needs, and were shared between clinics at the end of each intervention step during action learning sessions.^17^ Routine clinic registers were sourced to identify women eligible for HIV care tests during CQI activities, as antenatal medical records (maternity case records, MCRs) are retained by pregnant women until delivery. The same registers were accessed to track CQI progress using run charts.

###  Theory of Change

 As previously described,^49^ we hypothesized that this complex intervention would improve clinical processes through support provided by CQI mentors to health workers, which, supplemented by real-time data on performance, would enhance motivation. Collaborative root-cause analyses of clinical process gaps would enable health workers to identify and implement simple workable solutions. Health workers’ ability to continue routine clinical activities in parallel, as well as willingness and availability to participate in CQI were assumed.

###  Study Design

 To investigate the process of implementing CQI in this context we used convergent mixed methods,^52^ guided by Normalisation Process Theory (NPT)^53^ and the Tailored Implementation of Chronic Diseases (TICD) checklist.^54^

 NPT was developed to explain whether and how new technologies are taken up by healthcare teams.^53^ This framework allows investigation of how contextual factors such as resources, organisational culture and hierarchy, enable a new approach to be implemented into routine practice and thus ‘normalised.’ It suggests that successful integration requires 4 sorts of work for teams: (*i*) to make sense of the new approach (render it coherent); (*ii*) to maintain their own engagement with and delivery of the new approach and involve others (cognitive participation); (*iii*) to actually do the work of the new approach and ensure others are doing it (collective action); and (*iv*) monitor how well the approach is being implemented and thus its effectiveness (reflexive monitoring).^53^

 The TICD checklist was designed to identify determinants of practice to facilitate developing context-specific tailored interventions to improve healthcare quality. The checklist identifies factors that could influence uptake of interventions, organised in 7 broad domains: guideline factors, individual healthcare professional factors, patient factors, professional interactions, incentives and resources, capacity for organisational change, and social, political and legal factors.^54^

###  Study Setting 

 The study was located in the Hlabisa sub-district of KwaZulu-Natal, 220 km north of Durban, South Africa. HIV prevalence among women of reproductive age is ~37%.^47^ The local sub-district hospital, Hlabisa Hospital, oversees management of all 17 primary healthcare clinics in the area — this includes processing laboratory tests including VL, and provision of drug supplies. VL monitoring is routinely available at all clinics in the area. Clinics are nurse-led and receive medical officer support from Hlabisa Hospital about weekly. Clinical care is provided by professional nurses who are supervised by an operational manager (senior professional nurse). Professional nurses are assisted by lay counsellors (who perform HIV counselling and testing) and enrolled nurses. Data capturers are primarily responsible for capturing routine clinic data onto the national ART programme database, TIER.Net.

 The process evaluation was conducted at all 7 primary healthcare clinics participating in the MONARCH trial.

###  Data Sources

 We extracted data on ‘dose,’ ‘reach,’ how each clinic implemented CQI, and CQI mentor experiences of implementation from the CRH CQI mentor field notes. These notes detailed actual visit dates and type, attendance registers, root-cause analyses, improvement interventions (including start and review dates of PDSA cycles), and successes and challenges of implementation (including impressions of health worker understanding of eMTCT guidelines).

 We sourced descriptive primary endpoint data from medical records of women ≥18 years old at delivery.^49^

 Health worker experiences of implementing CQI were gathered from 15-30 minute semi-structured interviews conducted one month after the Intervention step at a particular clinic. We invited available health workers to interview, targeting those in leadership roles such as the operational manager where possible. Interviews were audio-recorded with consent and transcribed verbatim. The interview topic guide was informed by NPT (Supplementary file 1).

###  Data Analysis

 Our analysis proceeded in 5 steps. *First*, to understand ‘dose’ and ‘reach’ of CQI we summarised clinic size and setting, planned versus actual number of CQI meetings (‘dose’), and health worker participation in CQI meetings (‘reach’).

*Second*, to understand the ‘black box’ of implemented changes in practice, we summarised key root causes of poor HIV care testing and implemented change ideas.

*Third*, we summarised start and review dates of the first PDSA cycle as a proxy estimate of time to intervention uptake and assimilation. We then described each primary endpoint achievement by time step at each clinic, (time trends) to better understand the delayed CQI intervention effect we identified in our quantitative impact evaluation.^50,55^ The proportion of women living with HIV receiving a VL test ever in pregnancy, and proportion of women not living with HIV receiving a repeat HIV test ever in pregnancy, were estimated per clinic per time step. Women were allocated to a time step according to date of delivery. We assigned women to a particular clinic based on the first antenatal clinic visited.^50^

*Fourth*, we undertook a framework analysis of data from all CRH reports and field notes, to understand determinants of CQI implementation and ‘normalisation’ from the perspective of the CQI mentors. These documents were reviewed several times by one of the authors (HMY) to develop a coding frame: (*i*) knowledge and understanding of eMTCT guidelines; (*ii*) buy-in to CQI and willingness to acknowledge gaps in clinical practice; (*iii*) staff handover and communication; (*iv*) steps to identify and track women eligible for each test; (v) steps to follow up test results; (*vi*) routine data quality; (*vii*) availability of necessary supplies and space; (*viii*) availability of necessary healthcare personnel; and (*ix*) patient factors. This coding frame was applied across all collated documents to identify potential drivers of low HIV care testing prior to intervention start and factors influencing implementation. The codes were then organised using the TICD framework.^54^ How each factor may have influenced intervention delivery was also elucidated.

*Fifth*, transcripts from semi-structured interviews — to understand determinants of CQI implementation and ‘normalisation’ from the clinic health worker perspective — were reviewed several times (HMY), and a coding frame guided by NPT^53^ with additional emergent themes was developed. The overlapping codes included: (*i*) coherence (did health workers understand differences in CQI compared with old ways of working, did they understand, what they were expected to do, did they believe it might work); (*ii*) cognitive participation (were health workers willing to engage with the new activities, was there ownership); (*iii*) actual feasibility of implementing CQI and confidence in its potential (collective action); (*iv*) reflexive monitoring (impact on personal role, impact on patients, sustainability of CQI); (*v*) other contextual information including gaps in clinical processes; (*vi*) communicating with colleagues; (*vii*) tracking patients; (*viii*) insights on rationale for national guidelines on VL and repeat HIV testing; (*ix*) challenges implementing UTT. Barriers and facilitators of CQI implementation were then identified according to the TICD framework.^54^

 Finally, quantitative and qualitative data were summarised by clinic, using a mixed methods matrix.^56^

## Results

 We first present CQI participation by clinic health workers followed by the ‘black box’ of implemented changes. Second, we summarise time to PDSA cycle start and review, and variation in endpoint achievement by time step. Third, we present CQI mentor and health worker experiences of CQI implementation using the TICD framework. Finally, we provide an overview of the findings using a mixed methods matrix.

###  Health Worker Participation in CQI: “Dose” and “Reach”

 The main resource investment for CQI implementation was CQI mentor and clinic health workers’ time (Table 1 in our linked primary impact evaluation).^50^ All clinics participated in CQI activities after randomisation and staff were trained on all CQI tools. Almost all scheduled CQI meetings took place as planned, and all clinics utilised all CQI tools. Buy-in to CQI and the CRH team was strong with several clinics requesting additional visits (Table 2) — this resulted in a higher total ‘dose.’ Health worker participation in CQI visits (‘reach’) was less than anticipated, particularly among senior staff cadres (Table 3) — this was largely due to competing clinical commitments or staffing shortages (Table S3). Given challenges with attendance and staff turnover, training on CQI tools and PDSA cycles had to be repeated. Apart from expected contextual adaptation, the intervention was not modified.

**Table 2:** cheduled CQI Visits Versus Actual Visits Per Clinic (‘Dose’ of CQI), Over Entire Study

**Clinic** ^a^	**Type of Visit** ^b^				**Number of Action Learning Sessions**
	**Induction and Intervention Visits**		**Support and Maintenance Visits**		
	**Planned**	**Actual**	**Planned**	**Actual**	
All Clinics	79	**82**	163	**248**	3-7
Clinic 1	12	**17**	26	**56**	7
Clinic 2	11	**12**	24	**54**	7
Clinic 3a	11	10	22	**37**	6
Clinic 3b	11	9	22	**34**	6
Clinic 4	11	**14**	25	**29**	5
Clinic 5	11	8	23	**21**	4
Clinic 6	12	12	21	17	3

Abbreviation: CQI, continuous quality improvement. Meetings were conducted by CRH CQI mentors visiting facilities. ^a^Clinics are listed in order of randomisation. Clinics 3a and 3b were randomised to the same intervention step as they formed a single cluster. ^b^Extra visits were provided to clinics if requested by clinic health workers. Actual visits exceeding number of planned visits are highlighted in bold. Induction visits: visits conducted during the two-week lead-up to intervention rollover. These included situational analyses and training on CQI methodology. Intervention visits: visits conducted during the two-month intervention step using CQI tools to design and test solutions to drivers of low HIV care testing. Support visits: visits conducted during the two-month intervention step for additional support with using CQI tools, particularly reviewing changes in practice. Extra support visits were also provided after the intervention step, during the maintenance phase.^49^ Maintenance visits: visits conducted to consolidate skills learned. They were similar in function to support visits. Maintenance visits occurred after the intensive intervention step for each clinic and less frequently than intervention step support visits (about monthly).^49^ Clinics participated in action learning sessions based on their order of randomisation: clinics were invited to participate in these sessions immediately after randomisation (during the two-week CQI induction phase) or if they had already completed their CQI intervention step. There were seven action learning sessions held over the entire study.

**Table 3:** Summary of Clinic Health Worker Attendance (“Reach” Of CQI) at Clinic-Based CQI Meetings^d^

	**Number of Clinic Health Workers Participating in CQI**	**Full Clinic Health Worker Team Attended**		**Half Clinic Health Worker Team Attended**	**Only One Clinic Health Worker Attended**	**Professional Nurse Present**	**Lay Counsellor Present**	**Operational Manager Present** ^c^
		**All visits**	**Induction and Intervention Visits**	**All Visits**	**All Visits**	**All Visits**	**All Visits**	**All Visits**
All clinics	41	31/313 (10%)	14/79 (18%)	139/313 (44%)	55/313 (18%)	170/313 (54%)	119/205 (58%)	99/267 (37%)
Clinic 1	7	8/64^a^ (12%)	4/16^a^ (25%)	21/64^a^ (33%)	7/64^a^ (11%)	39/64^a^ (61%)	41/64^a^ (64%)	25/64^a^ (39%)
Clinic 2	6	3/62^a^ (5%)	2/10^a^ (20%)	27/62^a^ (44%)	12/62^a^ (19%)	40/62^a^ (64%)	NA^b^	31/62^a^ (50%)
Clinic 3a	4	4/46^a^ (9%)	0/10	26/46^a^ (56%)	16/46^a^ (35%)	25/46^a^ (54%)	NA^b^	NA^b^
Clinic 3b	6	6/42^a^ (14%)	2/9 (22%)	17/42^a^ (40%)	5/42^a^ (12%)	10/42^a^ (24%)	23/42^a^ (55%)	16/42^a^ (38%)
Clinic 4	6	3/41^a^ (7%)	1/14 (7%)	21/41^a^ (51%)	5/41^a^ (12%)	26/41^a^ (63%)	25/41^a^ (61%)	18/41^a^ (44%)
Clinic 5	6	3/29 (10%)	2/8 (25%)	15/29 (52%)	6/29 (21%)	17/29 (59%)	12/29 (41%)	1/29 (3%)
Clinic 6	6	4/29 (14%)	3/12 (25%)	12/29 (41%)	4/29 (14%)	13/29 (45%)	18/29 (62%)	8/29 (28%)

Abbreviations: CQI, continuous quality improvement; NA, not applicable. Attendance at visits was ascertained from meeting attendance registers. ^a^Denominator is different to actual total number of visits (Table 2) as some attendance registers were unavailable. ^b^Not applicable: staff member not recruited to clinic CQI team, or unavailable. ^c^In some instances, the operational manager may have attended meetings regardless of clinic CQI team membership. ^d^Proportion of initially recruited clinic health worker CQI team. Visits cover entire study period.

###  The “‘Black Box’” of Implemented Practice Changes 

 The CQI mentors trained all clinics on all CQI tools and utilised them consistently during implementation. At the beginning of each intervention step, root-cause analyses were conducted with process maps of patient flow and fishbone diagrams as part of a situational analysis at each clinic. There were discrepancies between monthly monitoring and evaluation (M&E) summary statistics and clinic registers, and incompleteness, suggesting problems with data quality (Supplementary file 1).

**Figure 1:**
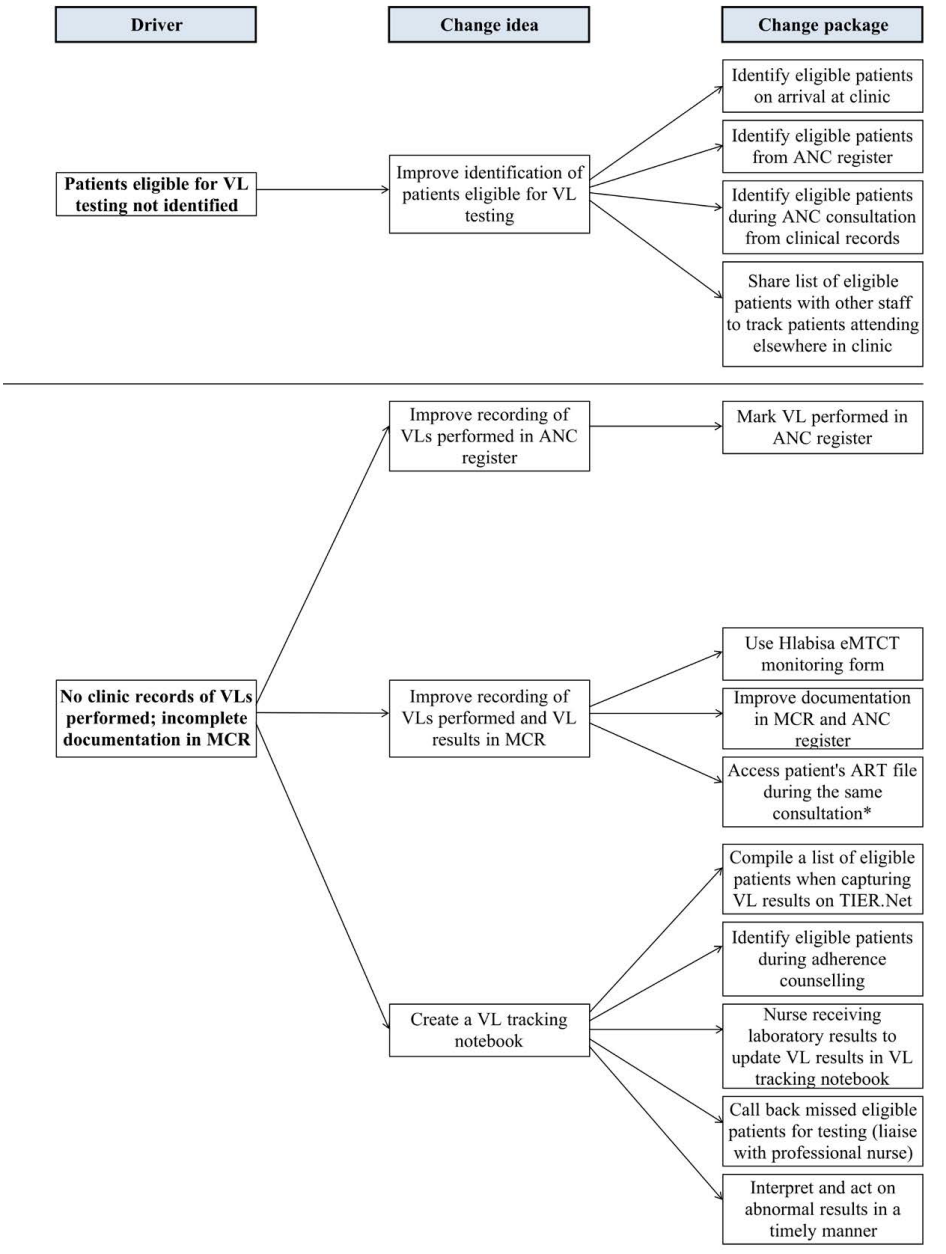
Drivers of Low VL Monitoring and Repeat HIV Testing Rates and Implemented Improvement Activities. **(a)** change ideas and package for improving VL monitoring; **(b)** change ideas and package implemented for improving repeat HIV testing; **(c) **change ideas and package implemented for other interventions including patient flow to streamline waiting times (therefore increased likelihood of patients being available for VL or HIV testing) and data quality. *All pregnant women living with HIV had a separate ART file based at the clinic, as did other individuals in the general ART programme. However, the MCR is retained by women until delivery and functions as a key communication modality between health workers particularly given that women may deliver at a facility other than their ANC clinic. Diagram format adapted from Osibo et al.^28^ Abbreviations: ANC, antenatal care; ART, antiretroviral therapy; eMTCT, elimination of mother-to-child transmission; MCR, maternity case record (antenatal medical record); VL, viral load.

 Each clinic had different organisational challenges (Table S1). There were differences in patient workload, building size, lay counsellor absence (one clinic), and computer breakdown (4 clinics). Common root causes — across most clinics — for low HIV care testing rates included poor clinical documentation, poor filing of VL results, and lack of a patient tracking system. Although the implemented change ideas (eg, patient tracking notebook for VL monitoring and repeat HIV testing) were similar (Figure 1), it was essential to adapt each solution to the clinic context. This resulted in heterogeneity of implementation.

###  Time to First PDSA Cycle Uptake and Time Trends in Endpoint Achievement

####  Timing of PDSA Cycle Reviews

 Table S2 summarises timing of the first PDSA cycle in relation to intervention rollover and review of the first PDSA cycle, by clinic. Larger clinics experienced delays in starting and/or reviewing (55-63 days) their first PDSA cycle whereas the smallest clinic with low clinical workload was able to rapidly start and review their improvement activities (5-7 days).

####  Descriptive Primary Endpoint Achievements by Time Step

 Medical records from 2160 women who delivered were analysed. HIV prevalence was 47%.^50^ Over the whole study, 56% women living with HIV had a VL performed ever in pregnancy (of whom 52% had a documented result) and 94% had an ART prescription. Repeat HIV testing among pregnant women not living with HIV was 67%.^50^

**Figure 2:**
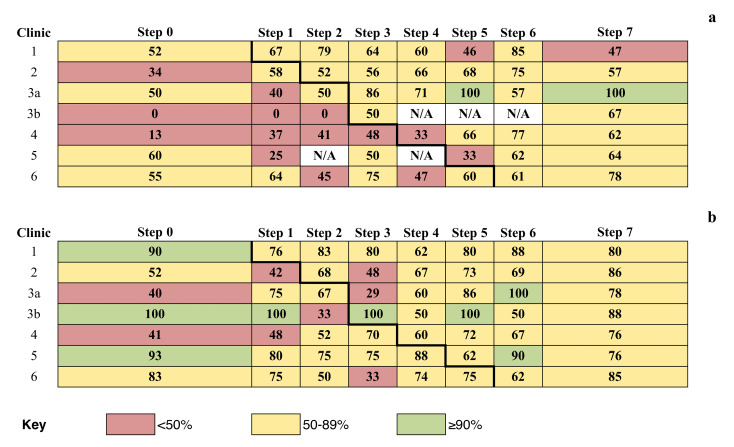
Proportion of Participants Achieving Primary Endpoints Ever During Pregnancy, Per Clinic Per Step.Thick black lines mark the point of rollover to the intervention**. (a) **proportion of women living with HIV who had a VL test ever in pregnancy;** (b) **proportion of women not living with HIV who had a repeat HIV test ever in pregnancy**. **Abbreviation: VL, viral load. N/A: no observations during that step. As clinics 3a and 3b had a small number of participants per step, their proportions should be interpreted with caution Clinics are listed in order of randomisation.

 There was an overall improvement in the HIV VL endpoint in post-intervention steps compared with pre-intervention steps (Figure 2a), consistent with our quantitative impact evaluation.^50^ There was considerable fluctuation in repeat HIV testing in post-intervention steps compared with pre-intervention. A higher pre-intervention repeat HIV testing rate was also evident (Figure 2b) and may explain the lack of CQI effect on improving repeat HIV testing in our quantitative impact evaluation.^50^

###  Determinants of CQI Implementation And “Normalisation”: CQI Mentor and Clinic Health Worker Experiences (TICD Framework)

 All clinics participated in process evaluation data collection. Semi-structured interviews with health workers lasted 15-60 minutes based on health worker availability at the time. One to 3 health workers were interviewed per clinic based on availability, totalling 16 interviews across all facilities: 2 operational managers, 4 professional nurses, 2 enrolled nurses, 3 data capturers, 2 nutrition advisors and 3 lay counsellors were interviewed.

 Below, we summarise CRH CQI mentor experiences (detailed in Table S3) and health worker interviews (detailed in Table S4).

####  Guidelines Factors

 Guidelines factors may be considered in 2 categories: South African national eMTCT guidelines, and the CQI intervention as a new practice ‘guideline.’

*South African National eMTCT Guidelines and Other Factors*: Because the focus of CQI was national eMTCT guidelines implementation, the guidelines were an enabler of the intervention. During the last few months of the MONARCH trial (September 2016), South Africa rolled out UTT,^46^ with likely knock-on effects on CQI throughout clinics due to increased demand on HIV services (Tables S3, S4).

 In March 2016, routine national Department of Health (DoH) M&E registers were overhauled for efficiency and the new registers no longer included an antenatal VL monitoring indicator: health workers had to depend entirely on the informal notebook created for patient tracking (Figure 1). Thus barriers to CQI activities included temporary disruptions during retraining on the new M&E registers and additional documentation for maintaining the HIV VL tracking notebook.

*CQI methodology as a ‘guideline’*: All interviewed health workers reported feeling supported by the CRH team and enjoyed working with them (Table S4). At the final action learning session several health workers expressed a wish to continue working with the CRH team after project end, as they felt they needed ongoing support. Visible improvements in practice (such as improved teamwork with identifying eligible women for HIV care tests) were also enablers of the intervention (Tables S3, S4). However, feasibility was challenged due to increased effort to implement improvement activities (such as better clinical documentation and implementing the patient tracking system), and incompatibility of routine DoH M&E registers with our primary endpoints although the latter were direct applications of national eMTCT guidelines. As a result, delays in implementing and/or reviewing the first PDSA cycle were noted at some clinics (Table S2).

####  Individual Health Professional Factors

 Key enablers of CQI implementation as reported by respondents, were buy-in to CQI methodology and motivation to improve quality of clinical services. All interviewed health workers found CQI methodology to be novel and interesting, and appreciated working with the CQI mentors. Some highlighted that CQI identified gaps in their current clinical practice and was an ‘eye-opener’ (Table S4). Although gaps in eMTCT domain knowledge were observed (Tables S1, S3), health workers reported better motivation to follow up patients once they understood the rationale of the guidelines (Table S4). However, challenges were many: limited self-efficacy from needing operational manager approval to implement any improvement activity, the need for repeated training and supervision to improve routine data quality, and poor clinical documentation in medical records (Tables S3, S4).

####  Patient Factors

 Respondents also identified patient needs and behaviours that contributed to CQI feasibility. Good patient rapport with health workers in the smaller more rural clinics (Table S3), and being proactive at following up results with their health worker (Tables S3, S4) may have facilitated better HIV care testing. However, some patients prematurely departed the clinic due to long queues, missing their HIV care tests (Tables S3, S4). Other patients’ mobile phones were inoperative or the number was incorrect, making it difficult to schedule follow-up visits. Health workers noted that many women first register for ANC later in pregnancy, making it difficult to conduct HIV care tests according to guidelines (Table S4). Finally, some patients were inflexible with changes to clinic workflow and lodged complaints (Table S3).

####  Professional Interactions

 Staff interactions were important for disseminating CQI skills and continuity of improvement activities. Enablers were teamwork and collegiality (Tables S3, S4). However, professional hierarchy hindered implementation even with good teamwork: it was difficult for lower cadre staff in some clinics to share new knowledge with their superiors, even though the former were more available to participate in CQI activities (Table S3). The operational manager was essential for decision-making to start a new activity (Tables S3, S4). Dissemination of CQI skills from clinic CQI team members to non-members was inconsistent. Data quality improvement and patient follow-up activities needed rigorous communication between different cadres of staff but were not implemented consistently during heavy clinical workload or staffing shortages. Communication and handover were additional challenges when an individual responsible for a particular activity went on leave (Table S3).

####  Incentives and Resources

 There were many resource constraints that likely impacted intervention implementation and general health service delivery. Most notable was staffing shortages in all clinics, particularly professional nurses — the shortages were worsened by resignations and 2 deaths (Table S3). As professional nurses are the main health workers providing ante- and post-natal care, their limited participation in CQI (Table 3) would have reduced translation of improvement activities to clinical care. Operational manager unavailability due to other commitments delayed approval of improvement activities. Opportunities for HIV retesting were missed during lay counsellor study leave – for example in one clinic ~120 eligible women not living with HIV did not receive repeat HIV tests over a 3-month period (Table S3). Additional constraints included limited building space, computer breakdown, and lack of printing equipment and essential clinical monitoring forms (Tables S1, S3). HIV test kit stockouts were observed in 4 clinics and ART stockouts in one (Table S3). One nurse highlighted the importance of hiring more lay counsellors as it was difficult to take on the additional responsibility of HIV testing and counselling with their existing workload (Table S4). These constraints also contributed to delays in implementing and/or reviewing PDSA cycles.

####  Capacity for Organisational Change

 The CQI mentors provided support for organisational change. However, limited operational manager availability may have reduced capacity for organisational change, as the operational manager was required to approve all activities. Professional hierarchy may have reduced individual health worker self-efficacy and ownership of change (Tables S3, S4**)**.

 Health workers overcame their initial resistance to change, given the need to improve the quality of patient care, and, as we have seen, in interviews were enthusiastic about CQI. While some believed CQI was sustainable, others highlighted that staff transfers to other facilities (resulting in loss of CQI memory at the facility) or lack of leadership to motivate a culture of quality, would reduce sustainability of CQI. Moreover, they believed an ongoing external stimulus from the CQI mentors or elsewhere (such as district supervisors) would be necessary to continue motivating them to continue CQI activities as CQI was not yet embedded in their practice; therefore the lack of such a stimulus was expected to negate progress in the future (Table S4).

####  Social and Political Factors

 In South Africa, lay counsellors have provided HIV counselling since 1995^57^ and HIV testing since 2010,^58^ predominantly funded by donors. To formally recognise lay counsellor employment status within the DoH, the KwaZulu-Natal DoH redeployed lay counsellors to different roles in 2015.^59,60^ As pointed out by one nurse, it was challenging to take on that extra work during lay counsellor absence (Table S4).

###  Determinants of CQI Implementation and “Normalisation”: Mixed Methods Matrix

 Fidelity of implementation was high from the side of the CQI mentors: all clinics were trained on all CQI tools and utilised them, the mentors adhered to the study timeline and minimum CQI dosage was exceeded. They also worked hard to encourage health worker participation in a supportive manner.

 Conversely, fidelity was lower from the side of clinic health workers. Health workers were enthusiastic about CQI and reported better motivation through understanding eMTCT guidelines. Key challenges influencing implementation included staffing shortages and turnover, data quality challenges, poor clinical documentation, needing operational manager approval for implementing CQI activities, and patient factors such as early departure from clinics due to long queues. There were additional resource constraints such as shortage of clinical monitoring forms and HIV test kits (Table 4).

**Table 4:** Mixed Methods Matrix of Factors Influencing Delivery and “Normalisation” of the CQI Intervention

**Quantitative**				**Qualitative**					
**Participation in CQI (% of All Visits)**	**Time to First PDSA** ^*^ ** Start **	**Time to First PDSA** ^*^ ** Review **	**Number of Steps to First Observed Improvement (≥10 Percentage Points)** ^§^	**Guidelines Factors**	**Individual Health Professional Factors**	**Patient Factors**	**Professional Interactions**	**Incentives and Resources**	**Capacity for Organisational Change**
**Clinic 1 (September 29, 2015)**									
***Medium size, rural setting***									
Operational manager 39%	2 days	20 days	*VL:*1 step	Staff turnover within clinic CQI team – affected trialability of intervention	Staff not familiar with 2015 eMTCT guidelines	Patients leave clinic prior to HIV care tests due to long queues	Limited sharing of CQI skills between clinic CQI team members and other clinic staff	Staffing shortages	Operational manager authorisation required to implement all CQI activities
Professional nurse 61%									
Lay counsellor 64%			*Rpt HIV test*: 0 steps						
					Limited self-efficacy	Patients not contactable for follow-up	Data quality challenges due to lack of communication between staff cadres	Poor documentation of tests and results in medical records	
					Data quality challenges due to limited understanding of M&E data	Patient not adherent to ART due to lack of food	Operational manager authorisation required to implement all CQI activities	DoH eMTCT monitoring forms not available	
								No printer cartridge for printing essential clinical and M&E forms	
								Landline out of order	
								Paper-based results and routine M&E	
**Clinic 2 (November 24, 2015)**									
***Large size, urban setting***									
Operational manager 50%	1 day	62 days	*VL: *4 steps	Staff turnover within clinic CQI team – affected trialability of intervention	Enthusiasm for CQI	Improved patient awareness of VL and voluntary attendance for results follow-up	Limited sharing of CQI skills between clinic CQI team members and other clinic staff	Staffing shortages	Operational manager authorisation required to implement all CQI activities
Professional nurse 64%									
Lay counsellor NA^**^			*Rpt HIV test: *1 step						
					Limited self-efficacy		Data quality challenges due to lack of communication between staff cadres	Paper-based results and routine M&E	
					No ownership of improvement activities		Operational manager authorisation required to implement all CQI activities	DoH eMTCT monitoring forms not available	
					Staff not familiar with 2015 eMTCT guidelines			HIV test kits out of stock	
**Clinic 3a (January 26, 2016)** ^‡^									
***Small size, rural setting***									
Operational manager NA^**^	86 days	70 days	*VL: *1 step		Limited self-efficacy	General clinic patients unwilling to adjust attendance to accommodate ANC patient needs	Data quality challenges due to lack of communication between staff cadres	Staffing shortages	Operational manager authorisation required to implement all CQI activities
Professional nurse 54%									
Lay counsellor NA^**^			*Rpt HIV test: *3 steps						
					Data quality challenges due to limited understanding of M&E data	Patients not contactable for follow-up	Operational manager authorisation required to implement all CQI activities	Poor documentation of tests and results in medical records	
					Staff not familiar with 2015 eMTCT guidelines			VL results delays	
								DoH eMTCT monitoring forms not available	
								Computer not working	
								Paper-based results and routine M&E	
**Clinic 3b (January 28, 2016)**									
***Very small size, rural setting***									
Operational manager 38%	7 days	5 days	*VL: *1 step		Staff not familiar with 2015 eMTCT guidelines	Clinic staff know community members very well due to living in deep rural community	Data quality challenges due to lack of communication between staff cadres	Staffing shortages	Operational manager authorisation required to implement all CQI activities
Professional nurse 24%									
Lay counsellor 55%			*Rpt HIV test: *1 step						
					Limited self-efficacy		Operational manager authorisation required to implement all CQI activities	Low clinical workload – more time to implement CQI activities	
					Data quality challenges due to limited understanding of M&E data		Good team spirit	Overcrowding on doctor’s day due to small clinic size	
							Difficult for lower cadre staff (eg, data capturer, lay counsellor) to feedback CQI information to more senior staff	DoH eMTCT monitoring forms not available	
								Poor documentation of tests and results in medical records	
								No printer cartridge for printing essential clinical and M&E forms	
								Paper-based results and routine M&E	
**Clinic 4 (March 17, 2016) ** ^†^									
***Large size, urban setting***									
Operational manager 44%	63 days	55 days	*VL: *2 steps	Staff turnover within clinic CQI team – affected trialability of intervention	Some staff not familiar with 2015 eMTCT guidelines		Good teamwork within clinic CQI team	Staffing shortages	Operational manager authorisation required to implement all CQI activities
Professional nurse 63%									
Lay counsellor 61%			*Rpt HIV test: *0 steps						
					Data quality challenges due to limited understanding of M&E data		Data quality challenges due to lack of communication between staff cadres	HIV test kits out of stock	
							Operational manager authorisation required to implement all CQI activities	Lack of space for sorting laboratory results	
								DoH eMTCT monitoring forms not available	
								Poor documentation of tests and results in medical records	
								Paper-based results and routine M&E	
**Clinic 5 (May 18, 2016)**									
***Small size, rural setting***									
Operational manager 3%	20 days	16 days	*VL: *2 steps		Limited self-efficacy	Demanding patients, also attend overnight even for non-emergencies	Limited sharing of CQI skills between clinic CQI team members and other clinic staff	Staffing shortages	Operational manager authorisation required to implement all CQI activities
Professional nurse 59%									
Lay counsellor 41%			*Rpt HIV test: *0 steps						
					Data quality challenges due to limited understanding of M&E data	Reluctance to queue for clinical consultations during daytime	Incomplete handover of patient tracking processes during periods of annual leave	HIV test kits out of stock	
							Data quality challenges due to lack of communication between staff cadres	ART out of stock	
							Operational manager authorisation required to implement all CQI activities	Poor documentation of tests and results in medical records	
								Paper-based results and routine M&E	
**Clinic 6 (July 19, 2016)**									
***Medium size, rural setting***									
Operational manager 28%	7 days	58 days	*VL: *2 steps		Limited self-efficacy		Incomplete handover of patient tracking processes during periods of annual leave	Staffing shortages	Operational manager authorisation required to implement all CQI activities
Professional nurse 45%									
Lay counsellor 62%			*Rpt HIV test: *2 steps						
					Data quality challenges due to limited understanding of M&E data		Data quality challenges due to lack of communication between staff cadres	HIV test kits out of stock	
							Operational manager authorisation required to implement all CQI activities		
**Health worker perspectives**									
				Increased awareness of eMTCT guidelines	Understanding rationale of eMTCT guidelines	Patients start ANC late in pregnancy	Good team work as a result of CQI	Staffing shortages	Resistance to change
				Increased effort needed to maintain patient tracking notebook as not compatible with M&E registers	Limited self-efficacy -needing operational manager for all decisions	Patients not contactable – cell phone not working	Needing leadership – operational manager to guide services and decisions	CQI interesting; CQI mentors nice people	CQI not sustainable without external mentorship or supervision
					CQI as an ‘eye opener’ on quality shortfalls				

Abbreviations: ANC, antenatal care; ART, antiretroviral therapy; CQI, continuous quality improvement; M&E, monitoring and evaluation; PDSA, Plan-Do-Study-Act cycle; eMTCT, elimination of mother-to-child transmission of HIV; VL, viral load. Clinic size is based on clinical workload rather than building size. Most participating clinics were in small single-storey buildings; DoH, Department of Health. Operational manager, professional nurse and lay counsellor participation as a proportion of all CQI visits at each clinic were estimated from attendance registers. ^*^ Time is in calendar days. PDSAs in this table refer to activities directly addressing HIV VL monitoring and/or repeat HIV testing (Figure 1, Change Ideas). General data quality improvement activities including other PDSAs (eg, checks for consistency between source documents) are not included in this table. ^**^Not applicable as health worker not recruited to clinic CQI team or not working at clinic. ^‡^ “Gross” staffing shortages noted at this clinic. ^†^ “Extreme” staffing shortages were noted at this clinic which was frequently full. The operational manager was on annual leave at the start of the intervention, and the Acting operational manager was often providing clinical services and unable to attend CQI meetings. ^§^Steps are counted from the step immediately preceding intervention rollover to the first noted improvement step (regardless of subsequent step trends) – eg, an improvement noted during the intervention rollover step was counted as 1 step to improvement. Clinics which had a decrease or minimal change throughout the post-intervention period were allocated 0 steps. Although the number of steps to first observed improvement is described, there were fluctuations in endpoint achievements with intermittent decline in testing as shown in Figure 2. Note: *Qualitative data* reported in this table are based on observations by the CRH CQI mentors and health worker interviews, listed according to the TICD framework. Details of each factor and its likely effect on intervention implementation are provided in Tables S3 and S4. *Quantitative data* summarise ‘reach’ of CQI for key health workers, time to first PDSA cycle start and review (proxy for time to intervention uptake), and number of time steps to first observed improvement^§^ in each endpoint (proxy for delayed intervention effect). Clinics are listed in order of randomisation with intervention rollover date in brackets.

Table 4 summarises factors influencing delivery and ‘normalisation’ of CQI by clinic. It shows that health worker participation in CQI (‘reach’) was limited, particularly among key staff. Many clinics were unable to immediately improve VL monitoring during the intervention time step according to the stepped-wedge design, and several clinics had no improvement in repeat HIV testing. There were delays in implementing the first PDSA cycle and/or review of the first PDSA cycle after implementation, particularly in larger clinics.

## Discussion

 We demonstrate that CQI can be an effective means of critical self-appraisal and process improvement in resource-limited nurse-led primary care clinics. The implemented workflow improvements were simple. Our findings complement our quantitative impact evaluation which found that CQI had a significant impact on improving HIV VL monitoring among pregnant women living with HIV, but did not improve repeat HIV testing among pregnant women not living with HIV.^50^ Although we expected that all pregnant women would receive the appropriate HIV care test at least once in pregnancy in this HIV hyperendemic setting, actual achievements fell well short. Despite health worker enthusiasm for CQI and the CQI mentors, and a higher-than-expected ‘dose’ of CQI, ‘reach’ was limited as clinic health workers had difficulty finding time to consistently attend meetings. Whilst implementation fidelity was high on the part of the CQI mentors, fidelity was lower from the side of the clinic health workers attributable to several health system limitations.

 There were many challenges which reduced ‘reach’ and fidelity of CQI implementation. Within the broader health system and local context, some issues were ‘fixed’ (such as small clinic buildings, paper-based M&E systems, general staffing shortages, gaps in routine data quality, and redeployment of lay counsellors). Superimposed on these were several short and medium-term ‘shocks’: the death of 2 nurses and resignations without replacement, an overhaul of the routine M&E system, and the rollout of UTT.

 Staffing shortages particularly influenced health worker availability to participate and engage in CQI meetings and improvement activities (reduced ‘reach’). Knowledge gaps of the content and rationale of 2015 eMTCT guidelines would have further delayed improvements. Unavailability of the operational manager delayed the decision to implement and review improvement activities. Challenges limiting health workers’ ability to identify patients eligible for HIV care tests included poor clinical documentation and routine data quality, and the paper-based M&E system (requiring rigorous documentation and communication between staff cadres). Poor data quality would have also reduced accuracy of estimated monthly HIV care testing targets and estimates of progress towards those targets. The ART stockouts highlight a broader health system issue prevalent in several sub-Saharan African countries.^61-64^ Given the staffing shortages, it is likely that many clinics in our study had difficulty undertaking proactive stocktaking to maintain continuous supplies. There may have been higher-level supply chain challenges as well, albeit outside the scope of our study. However, it is possible that supply chain management at all levels, is amenable to improvement initiatives as demonstrated elsewhere in sub-Saharan Africa.^65,66^ Many of these factors reflect the importance of adequate time and support for health workers to improve knowledge, skills, and maintain CQI activities.

 The lack of improvement in repeat HIV testing warrants additional consideration. Clinics were conducting repeat HIV testing of pregnant women at 32 weeks’ gestation following an HIV screen at first antenatal visit based on 2013 eMTCT guidelines (Table 1), resulting in relatively higher pre-intervention repeat HIV testing. Furthermore, HIV counselling and testing may have become embedded as an exclusively lay counsellor role prior to their redeployment, therefore other staff may have been reluctant to take on the task, as described during interviews. Patient characteristics such as lack of perceived risk,^67^ presenting for ANC late in pregnancy, or leaving the clinic early due to long queues may have also contributed. The period of HIV test kit stockouts, albeit brief, may have played a role.

 These different determinants of implementation would have had a different net effect on intervention fidelity at each clinic. Although some process improvements may have been immediate, comprehensive assimilation of CQI clinic-wide is likely to have taken time beyond the duration of our study if health workers remained committed and had time available. The health worker and CQI mentor reports indicate that CQI may not be normalised in this resource-limited context, without ongoing external support from CQI mentors, leadership, and ownership of change and investment in operational capacity. To further expand on the issue of leadership, clinic managers may benefit from onsite mentorship to enhance their advanced planning, problem solving, priority setting, and leadership and management skills as described in some studies.^68-70^ Such capacity building is likely to strengthen clinic operations — and health worker availability to participate in CQI activities regularly — whilst improving staff skills, morale, and patient health outcomes. This is achieved through shifting towards a more proactive approach to clinical service provision than a reactive approach.^68^

###  Comparison With Other Studies

 Our findings are supported by other published studies of CQI in sub-Saharan African primary care settings. A trial in Nigeria identified enthusiastic participation in CQI as a key driver of success.^28^ While patient waiting times were reduced with CQI, establishing new improvement processes including data quality, was slow. Similarly, a trial of CQI implementation in Malawi was challenged by staff turnover, lack of available leadership, and inadequate ongoing support.^27^ Both studies highlighted the need for extra time beyond the study schedule for full uptake of CQI. A quasi-experimental CQI study in Tanzania and Uganda experienced routine data quality challenges, shortage of medical supplies, and lack of health worker prioritisation of one endpoint.^71^

 Implementing CQI as part of the expanding eMTCT programme in South Africa and elsewhere has had successes and challenges.^72-75^ Three studies demonstrated improvements in ANC quality of care indicators,^72,74,75^ and one showed improved uptake of skilled health worker deliveries.^74^ However, another study encountered several challenges including resistance to CQI methodology and external CQI mentors (resulting in considerable time delays), and randomisation sometimes selecting out willing participants.^73^ The CQI mentors and methodology were very welcome in our study, perhaps because we had fewer participating facilities and held meetings at each clinic to introduce CQI before the trial began.

 Other CQI evaluations in non-primary care settings in sub-Saharan Africa highlight similar considerations. Despite good acceptance of the intervention, there were data quality challenges,^76^ poor roads during bad weather conditions affecting patient access to services,^76^ staffing shortages,^77^ high staff turnover,^77^ and patient-level barriers such as stigma.^77^

###  Strengths and Limitations 

 This study adds to the emerging evidence base for determinants of success in implementing CQI in resource-limited primary care settings. Conducting data collection in parallel to the main trial helped reduce measurement bias and optimised recall of health workers’ experiences. The complementary quantitative and qualitative data sources provided nuanced perspectives of CQI implementation. We were able to describe antenatal HIV health services in the area in detail with important implications for policy-makers. We were also able to obtain insights into time delays of CQI implementation, which may inform future research study designs.

 Our process evaluation had some limitations. We conducted final analyses and synthesis of data from the process evaluation after we knew the primary results of the stepped-wedge cluster RCT. Our interpretation of the data may have been different if we had conducted analyses for the impact and process evaluations separately. Although we identified features of the intervention indicative of whether it would be sustainable, we were unable to measure long term sustainability after the main trial ended. Measuring intervention sustainability is a crucial part of a study such as ours, given the interest in CQI by policy-makers elsewhere in South Africa,^72,78^ and is an important consideration for design of future studies. Finally, although implementation fidelity was high on the part of the CQI mentors, we did not measure the quality of their mentorship. It is possible that the quality of mentorship may not have been as high as expected and that the CQI mentors themselves needed additional training and supervision. However, given the close support by the improvement advisor during implementation,^50^ this is unlikely to have been a major issue.

###  Policy Implications

 Our process evaluation of CQI in resource-limited primary care settings demonstrates that it is possible to implement simple solutions to improve quality of services. The team of local CQI mentors, flexible with accommodating additional requests for support, were able to build and maintain rapport with health workers.

 Although the main resources investment for CQI is effort, prior to scaling up such a potentially desirable intervention, it is important to consider the intervention in the context of the broader health system. This is because there may be positive or detrimental effects on other services or components of the health system during scale up, including quality of other clinical services, funding, or other resources.

 Our findings on some of the health service limitations are also relevant to the goal of eMTCT. VL monitoring must improve substantially to (*i*) document actual ART response rather than rely on self-reported adherence; (*ii*) timely revise a failing ART regimen; (*iii*) optimise infant prophylaxis; and (*iv*) prevent onward transmission of HIV, particularly for women initiating ART later in pregnancy,^34,79-81^ and particularly given routine availability of the test. A recent systematic review identified that VL monitoring in resource-limited settings can be cost-effective if implemented judiciously and results acted upon.^82^ However, VL monitoring must be implemented alongside other interventions to facilitate and maintain VL suppression, including adherence to ART.^40^ Early diagnosis of incident HIV in pregnancy is also essential to allow early initiation of ART and VL suppression by delivery,^31,36^ therefore repeat HIV testing is essential to identify women who seroconvert during pregnancy. Finally, primary prevention of HIV should form part of the eMTCT programme, as demonstrated in countries that succeeded in eMTCT such as Cuba and Thailand.^41,42^

## Conclusions

 CQI is a flexible method for self-appraisal and process improvement in healthcare services. Despite health worker enthusiasm, staffing and other resource shortages limited ‘reach’ and fidelity of CQI in our study. CQI requires considerable support from the health system to realise its full potential in resource-limited settings.^3,83^ Strategies to empower junior staff to take ownership of change, better support from clinic managers, better communication between staff cadres, and efforts to improve routine data quality are important for maximal impact of improvement efforts. There is also an argument for reinstating lay counsellors or increasing nursing resource capacity in this setting. Moreover, designing routine M&E data collection instruments compatible with available clinical guidelines is critical to facilitate patient follow-up where electronic systems are not yet available.

 An important next step is to identify and rigorously test context-specific and sustainable solutions with a wide range of stakeholders, to address these health systems gaps — while considering the impact of the intervention on other components of the health system^84^ —to provide high quality healthcare services in resource-limited primary care settings.

## Acknowledgements

 The authors wish to thank the UKZN Centre for Rural Health for their efforts in implementing the CQI intervention, South African National Department of Health partners including primary health workers, and MONARCH study participants. We also extend our thanks to all colleagues at AHRI for their support with programme operations

## Ethical issues

 Ethical approval for the study was obtained from the University of KwaZulu-Natal Biomedical Research Ethics Committee (BREC, reference BE209/14). Approvals were obtained from district and sub-district level DoH staff prior to commencing the CQI intervention. Written informed consent was obtained from health workers prior to conducting semi-structured interviews. Standard DoH approvals for commencing the study were also obtained as part of a Memorandum of Understanding between AHRI and the Department of Health.

## Competing interests

 Authors declare that they have no competing interests.

## Authors’ contributions

 SW designed the process evaluation study in collaboration with TB, HMY, WDM, MM, and DP. HMY coordinated the main trial, performed the analysis and wrote the manuscript. HMY, WDM, MM, JWDN, CH, AJ, KP, FAP, DP, TB, and SW reviewed and edited the manuscript. TB is the principal investigator of the MONARCH trial. HMY and CH coordinated data collection for the process evaluation. All authors read and approved the final manuscript.

## Funding

 The MONARCH programme was co-funded by the Delegation of the European Commission to South Africa, EuropeAid/134286/L/ACT/ZA and the Wellcome Trust (through core funding to AHRI). The contents of this document are the sole responsibility of the authors and their affiliated institutions, and can under no circumstances be regarded as reflecting the position of the European Union.

 AHRI receives core funding from the UK Wellcome Trust grant 082384/Z/07/Z and Howard Hughes Medical Institute. The AHRI Population Intervention Platform is partially funded by the South African Population Research Infrastructure Network (SAPRIN), South African Department of Science and Technology.

 HMY is supported by an Australian Government Research Training Program (RTP) Scholarship, University of New South Wales, Sydney, Australia. The Kirby Institute is funded by the Australian Government Department of Health and Ageing, and is affiliated with the Faculty of Medicine, UNSW Sydney. JWDN is supported by the Alexander von Humboldt Foundation. TB is supported by the Alexander von Humboldt Professor award, funded by the Federal Ministry of Education and Research; the Wellcome Trust; and the NICHD of NIH (R01-HD084233), NIA of NIH (P01-AI112339), as well as FIC of NIH (D43-TW009775).

## Authors’ affiliations

^1^The Kirby Institute, University of New South Wales, Sydney, NSW, Australia. ^2^Africa Health Research Institute, KwaZulu-Natal, South Africa. ^3^School of Clinical Medicine, Discipline of Obstetrics and Gynaecology, University of KwaZulu-Natal, Durban, South Africa. ^4^School of Nursing and Public Health, University of KwaZulu-Natal, Durban, South Africa. ^5^Heidelberg Institute of Global Health (HIGH), Faculty of Medicine and University Hospital, University of Heidelberg, Heidelberg, Germany. ^6^King’s College Hospital NHS Foundation Trust, London, UK. ^7^Division of Infection and Immunity, University College London, London, UK. ^8^Department of Global Health and Population, Harvard T.H. Chan School of Public Health, Boston, MA, USA. ^9^Institute for Global Health, University College London, London, UK. ^10^Institute for Health & Wellbeing, University of Glasgow, Glasgow, UK.

## Supplementary files

Supplementary file 1 contains additional methods, semi-structured interview topic guide, and Tables S1-S4.
