# Supplementary material for: A Continuous Quality Improvement Intervention to Improve Antenatal HIV Care Testing in Rural South Africa: Evaluation of Implementation in a Real-World Setting
Source: Int J Health Policy Manag. 2020 Oct 27;11(5):610–28. doi: 10.34172/ijhpm.2020.178 (PMC9309927; doi:10.34172/ijhpm.2020.178)
Supplement: Supplementary file 1 — contains additional methods, semi-structured interview topic guide, and Tables S1-S4. [file ijhpm-11-610-s001.pdf]

Supplementary file 1

Additional file 1.

"A continuous quality improvement intervention to improve antenatal HIV care testing in rural South Africa: evaluation of implementation in a real-world setting"

This file includes:

- Additional methods
- Semi-structured interview topic guide
- Tables S1-S4

Data sources for CQI intervention at clinics

To estimate clinic-level performance on our primary endpoints in real-time, clinic registers containing routine Department of Health (DoH) monitoring and evaluation (M&E) indicators were sourced. Antenatal registers containing information on HIV testing, HIV results and treatment were accessed by the CRH team (CQI mentors) working with health workers at each clinic during improvement activities. Average monthly estimates of women eligible for each endpoint were made and set as targets for achievement. Monthly trends in actual testing were then plotted on run charts against the desired target for each endpoint. Additional data documents were created to implement patient follow-up and document results — these included an informal logbook (exercise book) per clinic with one staff member allocated as the main custodian. Data quality was poor with incompleteness and discrepancies between source registers and monthly summaries (summary statistics submitted to the district Facility Information Officer for collation at district, provincial and national level M&E). Poor clinical documentation in patient medical records was also noted.

Improvement activities were conducted to resolve routine data quality issues in M&E documents residing at clinics. However, as patient antenatal medical records (MCRs) were not available at clinics until after delivery, real-time improvement activities targeting clinical documentation were difficult to implement. Constraints of the stepped-wedge study design precluded comprehensive efforts at improving data quality as repeated training and supervision were needed.

Routine clinic registers and clinic-based CQI data sources were not accessed for the process evaluation or impact evaluation.

Topic Guide: Semi-structured interviews with staff

**General questions**	Can you tell me, overall, what has your experience been, working with the Quality Improvement team?<br>How did you find the education and training for QI?<br>How did you find getting feedback during PDSA cycles and Learning Sessions?<br>How did you find getting feedback from colleagues at other facilities during Learning Sessions? (NB: not applicable to clinic 1 until month 6 of study)
**NPT construct**	**Questions about the MONARCH QI intervention**
**Coherence:** Work to make sense of the system	How different is QI from 'old' ways of working?<br>Does everyone in the team share an understanding of its aims and what it is expected to deliver?<br>Does everyone in the team know what they are expected to do to deliver the system and how that differs from what they did before?<br>Do people really think the new system will work for them and will work to improve quality of care?
**Cognitive Participation:** Work to maintain their own engagement with and delivery of the new system and to involve others	How easy was it to get all concerned involved in implementing the new systems, including setting up systems and procedures (e.g. entering data in all DoH registers; creating run charts)?<br>Were people willing to invest time and effort in setting up the systems?<br>Do people see the new activities they need to do as their responsibility?<br>Do they know what they need to do to continue to make the system work?
**Collective Action:** The work that has to be carried out in practice in order to implement the system	How easy is it to complete routine tasks in this system?<br>Do people involved have the time (or flexibility in their schedule) to complete the tasks in this system?<br>Do people involved have the skills and training to undertake the required tasks?<br>Do they have confidence that they can do this and trust that it will work?
**Reflexive Monitoring:** The work that has to be done to monitor the system and to assess its effectiveness	How do those involved judge the effectiveness and/or success of the system?<br>What impact has the new system had on them and their roles?<br>Do they feel able to adapt how the system operates in the light of their experience of how it is working?

Table S1a. Summary of barriers to HIV VL monitoring at clinics[*] identified *prior* to receiving CQI intervention.

	Gaps in 2015 eMTCT guidelines knowledge	Poor recording in MCR of VL performed	No lay counsellor – increased workload for ANC nurse	Other staffing challenges despite lay counsellor present	VL sampled in ART department (rather than ANC)	VL results filing delays	No system for tracking eligible patients for testing, or follow-up of results
Clinic 1	√	√	-	√	-	√	√
Clinic 2	√	√	√	-	-	√	√
Clinic 3a	√	√	-	√	-	√	√
Clinic 3b	√	√	-	-	-	√	√
Clinic 4	√	√	-	√	-	√	√
Clinic 5	√	√	-	√	-	√	√
Clinic 6	√	-	-	-	√	-	-

	VL indicator no longer required in DHIS register (March 2016)	Patients leave clinic before VL test can be performed due to queues	Small clinic building	No printer cartridge or toner[#]	No eMTCT monitoring forms	Computer not working
Clinic 1	-	√	√	√	-	-
Clinic 2	-	N/A[§]	-	√	√	-
Clinic 3a	-	N/A[§]	√	√	√	√
Clinic 3b	-	N/A[§]	√	√	√	√
Clinic 4	√	√	√	√	-	√
Clinic 5	√	N/A[§]	-	-	N/A[§]	-
Clinic 6	√	-	-	-	N/A[§]	√

[*] Clinics are listed in order of randomisation

[#] Lack of printer cartridge or toner at clinics resulted in shortage of monthly tally sheets (for DoH monitoring and evaluation) – tally sheets were therefore supplied by Hlabisa Hospital

[§] Information not available

"√": identified as a barrier at that clinic; "-": not a barrier as clinic already had appropriate procedures or staff in place.

Barriers listed were those identified during situational analysis of each clinic (during two-week lead-in to Intervention step)

ANC, antenatal care; CQI, Continuous Quality Improvement; DHIS, District Health Information System; eMTCT, elimination of mother-to-child transmission of HIV; MCR, maternity case record; eMTCT, elimination of mother-to-child transmission of HIV; VL, HIV viral load.

Table S1b. **Summary of barriers to repeat HIV testing at clinics identified *prior* to receiving CQI intervention.**

	Gaps in 2015 eMTCT guidelines knowledge	Poor recording in MCR of HIV tests performed	No lay counsellor – increased workload for ANC nurse	Other staffing challenges	No system for tracking eligible patients for testing	Patients attend first ANC visit in 3rd trimester and do not return	Small clinic building	No printer cartridge or toner#
Clinic 1	√	-	-	√	√	N/A§	√	√
Clinic 2	√	-	√	√	√	√	-	√
Clinic 3a	√	-	-	√	√	N/A§	√	√
Clinic 3b	√	-	-	-	√	N/A§	√	√
Clinic 4	√	-	-	√	√	N/A§	√	√
Clinic 5	-	-	-	√	√	N/A§	-	-
Clinic 6	√	-	-	-	-	N/A§	-	√

* Clinics are listed in order of randomisation

Lack of printer cartridge or toner at clinics resulted in shortage of monthly tally sheets (for DoH monitoring and evaluation) – tally sheets were therefore supplied by Hlabisa Hospital

§ Information not available

"√": identified as a barrier at that clinic; "-": not a barrier as clinic already had appropriate procedures or staff in place.

Barriers listed were those identified during situational analysis of each clinic (during two-week lead-in to Intervention step).

ANC, antenatal care; DHIS, District Health Information System; eMTCT, elimination of mother-to-child transmission of HIV; MCR, maternity case record.

Table S2. Summary of first ever PDSA start dates and dates of first PDSA cycle review in relation to intensive Intervention rollover date, per clinic.

Clinic number, (size, setting)	Intensive Intervention start date	First PDSA start date	Review of first PDSA cycle		Time to PDSA start	Time to PDSA review (from PDSA start)
			Scheduled	Actual		
Clinic 1 (medium, rural)	29 September 2015	01 October 2015	07 October 2015	21 October 2015	2 days	20 days
Clinic 2 (large, urban)	24 November 2015	25 November 2015	08 December 2015	27 January 2016	1 day	62 days
Clinic 3a* (small, rural)	26 January 2016	21 April 2016	Not documented	30 June 2016	86 days	70 days
Clinic 3b (very small, rural)	28 January 2016	04 February 2016	11 February 2016	09 February 2016	7 days	5 days
Clinic 4** (large, urban)	17 March 2016	19 May 2016*	Not documented	13 July 2016	63 days	55 days
Clinic 5 (small, rural)	18 May 2016	07 June 2016	14 June 2016	23 June 2016	20 days	16 days
Clinic 6 (medium, rural)	19 July 2016	26 July 2016	09 August 2016	22 September 2016	7 days	58 days

* "Gross" staffing shortages at clinic 3a; delays implementing PDSA likely due to unavailability of operational manager +/- professional nurse to kickstart activities

** "Extreme" staffing shortages were noted at this clinic which was frequently full. The operational manager was on annual leave at the start of the intervention, and the Acting operational manager was often providing clinical services and unable to attend CQI meetings.

PDSA dates refer to activities relevant to HIV VL monitoring and/or repeat HIV testing (Figure 1, Change Ideas). General data quality improvement activities including PDSAs (e.g. checks for consistency between source documents) are not included in this table.

PDSA, Plan-Do-Study-Act cycle.

Table S3. Summary of factors influencing delivery and 'normalisation' of the intervention as reported by the CQI mentors (CRH): Tailored Implementation of Chronic Diseases (TICD) framework.

Clinic number (intervention start date)	TICD framework category	Observation	Reason(s)	How intervention delivery may have been influenced
**Clinic 1 (29 Sep 2015)**				
	Capacity for organisational change: mandate; leadership; regulations<br>*Individual health professional factors:* cognitions (self-efficacy)<br>*Professional interactions:* communication and influence	• Operational manager frequently absent from CQI meetings	• Sickness<br>• Competing priorities: district DoH meetings, clinical duties	• Operational manager (leadership) presence required for decision making and ownership of improvement activities
	Incentives and resources: availability of necessary resources	• Staffing shortages	• Pre-existing shortages<br>• One professional nurse resigned 2 months after study start<br>• Two professional nurses died in April/ May 2016<br>• Pay day each month – many staff not on duty	• Difficult for staff on duty to find time to attend CQI meetings<br>• Difficult for staff on duty to find time to implement improvement activities<br>• Staff turnover slowed uptake of CQI skills and consistency of implementation
		• DoH eMTCT monitoring forms not available	• Delay in arrival of stock from district hospital by >6 months	• Missed opportunities for testing patients eligible for VL monitoring

Clinic number (intervention start date)	TICD framework category	Observation	Reason(s)	How intervention delivery may have been influenced
		• No printer cartridge available	• Delays in procurement of replacement cartridge	• Difficult to implement data quality improvement activities
		• Unable to telephone patients identified for tracing	• Landline out of order	• Missed opportunities for testing patients eligible for VL monitoring and repeat HIV testing
	Professional interactions: team processes	• Limited sharing of CQI skills between clinic CQI team members and other clinic staff	• Only some clinic staff could attend CQI meetings given need to continue routine clinical activities in parallel<br>• Clinic CQI team selected by operational manager at start of intervention step<br>• Lack of time to formally share CQI tools and disseminate improvement activities	• Delayed implementation of improvement activities throughout clinic
	Professional interactions: team processes<br><br>*Guidelines factors**: recommended behaviour (trialability)	• Staff turnover within clinic CQI team	• Staffing shortages<br><br>• Non- clinic CQI team staff keen to participate in CQI by direct interaction with CRH team	• Delayed implementation of improvement activities due to need for repeated training and progress updates<br>• Better awareness of CQI tools throughout clinic at end of project
	Individual health professional factors: knowledge and skills	• Staff not familiar with changes in guidelines for HIV VL monitoring and repeat HIV testing	• Insufficient training on 2015 eMTCT guidelines	• Missed opportunities for testing patients eligible for VL monitoring and repeat HIV testing

Clinic number (intervention start date)	TICD framework category	Observation	Reason(s)	How intervention delivery may have been influenced
	Individual health professional factors: knowledge and skills; cognitions; professional behaviour<br><br>*Professional interactions:* team processes<br><br>*Incentives and resources:* information system	• Routine data quality challenges	• Inconsistent application of data quality improvement activities – repeated training and supervision needed<br>• Communication between different cadres of staff (each responsible for completing different source documents) needed<br>• Poor documentation of tests performed and test results in source documents (patient medical records, clinic registers etc)<br>• Paper-based systems with multiple sources of information	• Missed opportunities for testing patients eligible for VL monitoring and repeat HIV testing<br>• Unreliable estimates of monthly testing targets for VL monitoring and repeat HIV testing<br>• Unreliable estimates of progress towards monthly targets
	Patient factors: needs; knowledge; motivation; behaviours.	• Patients leave clinic before HIV VL test or repeat HIV test	• Long queues	• Missed opportunities for testing patients eligible for VL monitoring and repeat HIV testing
		• Patients not attending scheduled follow-up visit for HIV VL monitoring or repeat HIV testing	• Not answering phone<br>• Incorrect phone number provided<br>• Contacted but do not attend scheduled appointment	• Missed opportunities for testing patients eligible for VL monitoring and repeat HIV testing

Clinic number (intervention start date)	TICD framework category	Observation	Reason(s)	How intervention delivery may have been influenced
		• Patient with elevated HIV VL ~3500 copies/mL	• Not taking ART regularly as no regular food<br>• Vomits if takes ART without food<br>• No income	• Risk of MTCT of HIV and compromised maternal health<br>•<br>• Risk of ART drug resistance given viral replication in presence of drug selection pressure
**Clinic 2 (24 Nov 2015)**				
	Capacity for organisational change: mandate; leadership; regulations<br>*Individual health professional factors*: cognitions (self-efficacy)<br>*Professional interactions:* communication and influence	• Operational manager frequently absent from CQI meetings	• Competing priorities: district DoH meetings, clinical duties	• Operational manager (leadership) presence required for decision making and ownership of improvement activities
	Individual health professional factors: cognitions (attitudes)	• Staff would love to be involved in CQI	• Prospect of improving service areas	• Improved motivation to participate in CQI
	Incentives and resources: availability of necessary resources	• Staffing shortages	• Pre-existing shortages<br>• Several staff on annual leave December 2015-January 2016	• Difficult for staff on duty to find time to attend CQI meetings<br>• Difficult for staff on duty to find time to implement improvement activities

Clinic number (intervention start date)	TICD framework category	Observation	Reason(s)	How intervention delivery may have been influenced
			• 2 professional nurses retired<br>• 2 professional nurses resigned	
		• Delay identifying eligible patients for HIV VL monitoring and repeat HIV testing (from clinic registers, monthly summaries, during capturing of VL results)	• DC very busy due to competing clinical priorities	• Missed opportunities for testing patients eligible for VL monitoring and repeat HIV testing<br>• Difficulty finding time to implement improvement activities
		• DoH eMTCT monitoring forms not available	• No printer cartridge available for photocopying	• Missed opportunities for testing patients eligible for VL monitoring
		• VL results not filed on time<br>• VL tracking notebook not completed	• ANC clinic rooms flooded due to heavy rains and inaccessible for VL results follow-up and filing	• Risk of MTCT if elevated HIV VL<br>• Missed opportunities for testing patients eligible for VL monitoring
		• HIV test kits out of stock	• Delays in procurement	• Missed opportunities for testing patients eligible for repeat HIV testing<br>• Delays in maternal HIV diagnosis and treatment
	Professional interactions: team processes	• Limited sharing of CQI skills between clinic CQI team members and other clinic staff	• Only some clinic staff could attend CQI meetings given need to continue routine clinical activities in parallel	• Delay in implementation of improvement activities throughout clinic

Clinic number (intervention start date)	TICD framework category	Observation	Reason(s)	How intervention delivery may have been influenced
			• Clinic CQI team selected by operational manager at start of intervention step<br>• Two professional nurses participating in clinic CQI team very motivated, but when absent CQI implementation varied<br>• Lack of time to formally share CQI tools and disseminate improvement activities	
	Professional interactions: team processes	• Staff turnover within clinic CQI team	• Staffing shortages	• Delayed implementation of improvement activities due to need for repeated training and progress updates
		• Duplicate documentation of testing rates among eligible women (August 2016)	• NGOs supporting VL monitoring and repeat HIV testing had separate logbooks	• Unreliable estimates of monthly testing targets for HIV VL or repeat HIV testing<br>• Unreliable estimates of progress towards monthly targets
	Professional interactions: team processes<br>*Individual health professional factors:* cognitions	• Lack of ownership of improvement activities	• Staff turnover within clinic CQI team<br>• Operational manager unavailable to lead decisions and improvement activities	• Delayed implementation of improvement activities<br>• Missed opportunities for testing patients eligible for VL monitoring and repeat HIV testing

Clinic number (intervention start date)	TICD framework category	Observation	Reason(s)	How intervention delivery may have been influenced
	Individual health professional factors: knowledge and skills; cognitions	• Gaps in knowledge of guidelines criteria for HIV VL monitoring	• Insufficient training on 2015 eMTCT guidelines	• Missed opportunities for testing patients eligible for VL monitoring<br>• Inappropriately timed HIV VL monitoring
	Individual health professional factors: knowledge and skills; cognitions; professional behaviour<br><br>*Professional interactions:* team processes<br><br>*Incentives and resources:* information system	• Routine data quality challenges	• Inconsistent application of data quality improvement activities – repeated training and supervision needed<br>• Communication between different cadres of staff (each responsible for completing different source documents) needed<br>• Poor documentation of tests performed and test results in source documents (patient medical records, clinic registers etc)<br>• Paper-based systems with multiple sources of information	• Missed opportunities for testing patients eligible for VL monitoring and repeat HIV testing<br>• Unreliable estimates of monthly testing targets for HIV VL or repeat HIV testing<br>• Unreliable estimates of progress towards monthly targets
	Patient factors: knowledge; motivation; behaviour	• Improved patient awareness of VL —some patients return to follow-up results	• Improved patient education by healthcare providers	• Improved follow-up of results and timely management of virologic failure
**Clinic 3a (26 Jan 2016)**				

Clinic number (intervention start date)	TICD framework category	Observation	Reason(s)	How intervention delivery may have been influenced
	Capacity for organisational change: mandate; leadership; regulations<br><br>*Individual health professional factors*: cognitions (self-efficacy)<br><br>*Professional interactions:* communication and influence	• Operational manager did not join clinic CQI team	• Competing priorities: district DoH meetings, clinical duties	• Operational manager (leadership) presence required for decision making and ownership of improvement activities
	Incentives and resources: availability of necessary resources	• Staffing shortages	• Pre-existing shortages<br><br>• ART professional nurse resigned	• Difficult for staff on duty to find time to attend CQI meetings<br>• Difficult for staff on duty to find time to implement improvement activities
		• Computer not working March-November 2016	• Delayed repairs	• Backlog in capturing routine data including VL results on TIER.Net<br>• Missed opportunities for testing patients eligible for VL monitoring
		• DoH eMTCT monitoring forms not available	• Delay in arrival of stock from district hospital	• Missed opportunities for testing patients eligible for VL monitoring
		• HIV VL results not available in a timely manner	• Delays in HIV VL results dispatch from district hospital	• Missed opportunities for managing virologic failure in a timely manner

Clinic number (intervention start date)	TICD framework category	Observation	Reason(s)	How intervention delivery may have been influenced
				• Missed opportunities for testing patients eligible for VL monitoring
	Individual health professional factors: cognitions (self-efficacy)	• HIV VL and repeat HIV testing tracking notebook not used for 3 months	• HIV VL and repeat HIV testing tracking notebook ceased by district staff June-August 2016<br>• Clinic staff not empowered to justify its ongoing use to district-level staff	• Missed opportunities for testing patients eligible for VL monitoring and repeat HIV testing<br>• Unreliable estimates of monthly testing targets for HIV VL or repeat HIV testing<br>• Unreliable estimates of progress towards monthly targets
	Individual health professional factors: knowledge and skills	• Staff not familiar with changes in guidelines for HIV VL monitoring and repeat HIV testing	• Insufficient training on 2015 eMTCT guidelines	• Missed opportunities for testing patients eligible for VL monitoring and repeat HIV testing
	Individual health professional factors: knowledge and skills; cognitions; professional behaviour<br>*Professional interactions:* team processes<br>*Incentives and resources:* information system	• Routine data quality challenges	• Inconsistent application of data quality improvement activities – repeated training and supervision needed<br>• Communication between different cadres of staff (each responsible for completing different source documents) needed<br>• Poor documentation of tests performed and test results in source documents (patient	• Missed opportunities for testing patients eligible for VL monitoring and repeat HIV testing<br>• Unreliable estimates of monthly testing targets for HIV VL or repeat HIV testing<br>• Unreliable estimates of progress towards monthly targets

Clinic number (intervention start date)	TICD framework category	Observation	Reason(s)	How intervention delivery may have been influenced
			medical records, clinic registers etc)<br>• Paper-based systems with multiple sources of information	
		• HIV counselling and testing (general clinic) register not updated with patient's pregnancy status	• NGO assisting clinic with HIV testing (September 2016) was not documenting pregnancy status in HIV counselling and testing register	• Missed opportunities for testing patients eligible for repeat HIV testing<br>• Unreliable estimates of monthly testing targets for repeat HIV testing<br>• Unreliable estimates of progress towards monthly targets
	Patient factors: needs; knowledge; motivation; behaviours.	• Rearrangement of antenatal patient clinic times to early morning (PDSA cycle to streamline workflow) unsuccessful	• General clinic patients unwilling to attend clinic later in the morning – complaint lodged to Health Committee	• Difficult for staff on duty to find time to implement improvement activities
		• Patients not traceable	• Incorrect phone number provided	• Missed opportunities for testing patients eligible for VL monitoring and repeat HIV testing
**Clinic 3b (28 Jan 2016)**				
	Capacity for organisational change: mandate; leadership; regulations	• Operational manager frequently absent from CQI meetings	• Competing priorities: district DoH meetings, clinical duties	• Operational manager (leadership) presence required for decision making and

Clinic number (intervention start date)	TICD framework category	Observation	Reason(s)	How intervention delivery may have been influenced
	Individual health professional factors: cognitions (self-efficacy)<br><br>*Professional interactions:* communication and influence			ownership of improvement activities
	Incentives and resources: availability of necessary resources	• Staffing shortages	• Pre-existing shortages	• Difficult for staff on duty to find time to attend CQI meetings
		• Improvement activities completed quickly	• Lower clinical workload compared with other clinics	• Easier to complete improvement activities
		• DoH eMTCT monitoring forms not available	• Delay in arrival of stock from district hospital	• Missed opportunities for testing patients eligible for VL monitoring
		• No printer cartridge available to print tally sheets or data charts	• Delays in procurement of replacement cartridge	• Difficult to implement data quality improvement activities
		• Overcrowding during doctor's day at clinic	• Small clinic<br><br>• Extra patient turnout for Old Age Pension authorisation	• Possible missed opportunities for testing patients eligible for VL monitoring and repeat HIV testing<br><br>• Difficult for staff on duty to find time to implement improvement activities
		• Busy clinic during Ideal Clinic assessment preparation	• Nationwide DoH project to improve quality of primary care entitled "Ideal Clinic"	• Difficult for staff on duty to find time to attend CQI meetings<br><br>• Difficult for staff on duty to find time to implement improvement activities

Clinic number (intervention start date)	TICD framework category	Observation	Reason(s)	How intervention delivery may have been influenced
	Professional interactions: team processes	• Good team work and team spirit	• Small clinic	• Easier to complete improvement activities
	Professional interactions: team processes; communication and influence	• Ill feelings when lower cadre staff (e.g. data capturer, lay counsellor) feedback new information to more senior staff in clinic	• More senior staff (professional nurses, operational manager) unable to attend CQI meeting, therefore lower cadre staff provide updates<br>• Strong hierarchy in clinic	• Difficult to disseminate new information<br>• Delayed implementation of improvement activities<br>• May impact on sustainability
	Individual health professional factors: knowledge and skills; cognitions; professional behaviour<br><br>*Professional interactions:* team processes<br><br>*Incentives and resources:* information system	• Routine data quality challenges	• Inconsistent application of data quality improvement activities – repeated training and supervision needed<br>• Communication between different cadres of staff (each responsible for completing different source documents) needed<br>• Poor documentation of tests performed and test results in source documents (patient medical records, clinic registers etc)<br>• Paper-based systems with multiple sources of information	• Missed opportunities for testing patients eligible for VL monitoring and repeat HIV testing<br>• Unreliable estimates of monthly testing targets for HIV VL or repeat HIV testing<br>• Unreliable estimates of progress towards monthly targets

Clinic number (intervention start date)	TICD framework category	Observation	Reason(s)	How intervention delivery may have been influenced
	Individual health professional factors: knowledge and skills; cognitions	• Gaps in knowledge of guidelines criteria for HIV VL monitoring at first ANC visit (for pregnant women who are HIV-positive)	• Insufficient training on 2015 eMTCT guidelines	• Missed opportunities for testing patients eligible for VL monitoring<br>• Inappropriately timed VL monitoring
	Patient factors: needs; behaviours.	• Clinic healthcare providers know community members very well	• Deep rural clinic, many healthcare providers live in the same community as clients	• Improved patient tracking and tracing
**Clinic 4 (17 Mar 2016)**				
	Capacity for organisational change: mandate; leadership; regulations<br>*Individual health professional factors:* cognitions (self-efficacy)<br>*Professional interactions:* communication and influence	• Operational manager frequently absent from CQI meetings	• Competing priorities: district DoH meetings, clinical duties	• Operational manager (leadership) presence required for decision making and ownership of improvement activities
	Incentives and resources: availability of necessary resources	• Staffing shortages	• Pre-existing shortages<br>• Antenatal professional nurse on maternity leave<br>• Increased demand on clinical services with nationwide rollout of Universal Test and Treat (UTT) September 2016	• Difficult for staff on duty to find time to attend CQI meetings<br>• Difficult for staff on duty to find time to implement improvement activities<br>• Staff turnover slowed uptake of CQI skills and consistency of implementation

Clinic number (intervention start date)	TICD framework category	Observation	Reason(s)	How intervention delivery may have been influenced
		• DoH eMTCT monitoring forms not available	• Delay in arrival of stock from district hospital	• Missed opportunities for testing patients eligible for VL monitoring
		• HIV test kits out of stock	• Delays in procurement	• Missed opportunities for testing patients eligible for repeat HIV testing<br>• Delays in maternal HIV diagnosis and treatment
		• Limited space for sorting laboratory results	• Small clinic building	• Missed opportunities for testing patients eligible for HIV VL<br>• Difficult for staff on duty to find time to implement improvement activities
	Professional interactions: team processes	• Staff turnover within clinic CQI team	• Staffing shortages	• Delayed implementation of improvement activities due to need for repeated training and progress updates
	Professional interactions: team processes	• Good teamwork identifying eligible patients for VL monitoring and repeat HIV testing; bringing ART files to antenatal clinic	• Good staff motivation to improve quality of patient care	• Easier to complete improvement activities
	Individual health professional factors: knowledge and skills; cognitions; professional behaviour	• Routine data quality challenges	• Inconsistent application of data quality improvement activities – repeated training and supervision needed	• Missed opportunities for testing patients eligible for VL monitoring and repeat HIV testing

Clinic number (intervention start date)	TICD framework category	Observation	Reason(s)	How intervention delivery may have been influenced
	Professional interactions: team processes		• Communication between different cadres of staff (each responsible for completing different source documents) needed	• Unreliable estimates of monthly testing targets for HIV VL or repeat HIV testing
	Incentives and resources: information system		• Poor documentation of tests performed and test results in source documents (patient medical records, clinic registers etc)<br>• Paper-based systems with multiple sources of information	• Unreliable estimates of progress towards monthly targets
		• HIV counselling and testing (general clinic) register not updated with patient's pregnancy status	• NGO assisting clinic with HIV testing (September 2016) was not documenting pregnancy status in HIV counselling and testing register	• Missed opportunities for testing patients eligible for repeat HIV testing<br>• Unreliable estimates of monthly testing targets for repeat HIV testing<br>• Unreliable estimates of progress towards monthly targets
	Individual health professional factors: knowledge and skills; cognitions	• Decrease in repeat HIV testing after main professional nurse went on maternity leave	• Other professional nurse was including HIV tests performed in a previous pregnancy<br>• Insufficient training on 2015 eMTCT guidelines	• Missed opportunities for testing patients eligible for repeat HIV testing
**Clinic 5 (18 May 2016)**				

Clinic number (intervention start date)	TICD framework category	Observation	Reason(s)	How intervention delivery may have been influenced
	Capacity for organisational change: mandate; leadership; regulations<br>*Individual health professional factors:* cognitions (self-efficacy)<br>*Professional interactions:* communication and influence	• Operational manager frequently absent from CQI meetings	• Competing priorities: district DoH meetings, clinical duties	• Operational manager (leadership) presence required for decision making and ownership of improvement activities
	Incentives and resources: availability of necessary resources	• Staffing shortages	• Pre-existing shortages<br>• Increased demand on clinical services with nationwide rollout of Universal Test and Treat (UTT) September 2016<br>• Several staff on annual leave December 2016 -January 2017	• Difficult for staff on duty to find time to attend CQI meetings<br>• Difficult for staff on duty to find time to implement improvement activities<br>• Staff turnover slowed uptake of CQI skills and consistency of implementation
		• HIV test kits out of stock	• Delays in procurement	• Missed opportunities for testing patients eligible for repeat HIV testing<br>• Delays in maternal HIV diagnosis and treatment
		• ART out of stock	• Delays in procurement	• Delays in initiating maternal ART<br>• Risk of MTCT of HIV and compromised maternal health

Clinic number (intervention start date)	TICD framework category	Observation	Reason(s)	How intervention delivery may have been influenced
				• Risk of ART drug resistance if break in already initiated treatment
		• MCRs out of stock	• Not recorded	• Missed opportunities for clinical communication between ANC facility and delivery site including HIV status and treatment history
	Professional interactions: team processes; communication	• Period of missed documentation (in HIV VL and repeat HIV testing notebook) of HIV VL performed and results, prior to results filing	• Staff member in charge of this task went on annual leave without handover to colleagues	• Missed opportunities for testing patients eligible for VL monitoring and repeat HIV testing
	Professional interactions: team processes	• Limited sharing of CQI skills between clinic CQI team members and other clinic staff	• Only some clinic staff could attend CQI meetings given need to continue routine clinical activities in parallel<br>• Clinic CQI team selected by operational manager at start of intervention step<br>• Lack of time to formally share CQI tools and disseminate improvement activities	• Delay in implementation of improvement activities throughout clinic
	Individual health professional factors: knowledge and skills; cognitions; professional behaviour	• Routine data quality challenges	• Inconsistent application of data quality improvement activities – repeated training and supervision needed	• Missed opportunities for testing patients eligible for VL monitoring and repeat HIV testing

Clinic number (intervention start date)	TICD framework category	Observation	Reason(s)	How intervention delivery may have been influenced
	Professional interactions: team processes		• Communication between different cadres of staff (each responsible for completing different source documents) needed	• Unreliable estimates of monthly testing targets for HIV VL or repeat HIV testing
	Incentives and resources: information system		• Poor documentation of tests performed and test results in source documents (patient medical records, clinic registers etc)<br>• Paper-based systems with multiple sources of information	• Unreliable estimates of progress towards monthly targets
	Patient factors: needs; behaviours.	• Patients (including general clinic patients) demanding and attend overnight even for non-emergencies	• Community aware of nurse on duty overnight, although same nurse continues working throughout the following day<br>• Reluctance to stay in queues during the day	• Difficult for staff on duty to find time to attend CQI meetings<br>• Difficult for staff on duty to find time to implement improvement activities
**Clinic 6 (19 July 2016)**				
	Capacity for organisational change: mandate; leadership; regulations<br>*Individual health professional factors:* cognitions (self-efficacy)	• Operational manager frequently absent from CQI meetings	• Competing priorities: district DoH meetings, clinical duties<br>• Retirement	• Operational manager (leadership) presence required for decision making and ownership of improvement activities

Clinic number (intervention start date)	TICD framework category	Observation	Reason(s)	How intervention delivery may have been influenced
	Professional interactions: communication and influence			
	Incentives and resources: availability of necessary resources	• Staffing shortages: gross	• Pre-existing shortages<br>• Several staff on annual leave December 2016 -January 2017<br>• Lay counsellor retraining as pharmacy assistant<br>• Two professional nurses resigned<br>• Increased demand on clinical services with nationwide rollout of Universal Test and Treat (UTT) September 2016	• Difficult for staff on duty to find time to attend CQI meetings<br>• Difficult for staff on duty to find time to implement improvement activities<br>• Staff turnover slowed uptake of CQI skills and consistency of implementation
		• HIV test kits out of stock	• Delays in procurement	• Missed opportunities for testing patients eligible for repeat HIV testing<br>• Delays in maternal HIV diagnosis and treatment
	Professional interactions: team processes; communication	• 120 eligible patients for HIV testing missed during 3-month period	• Lay counsellor sole custodian of HIV VL and HIV testing notebook<br>• Lay counsellor in charge of repeat HIV testing	• Missed opportunities for testing patients eligible for repeat HIV testing<br>• Delays in maternal HIV diagnosis and treatment

Clinic number (intervention start date)	TICD framework category	Observation	Reason(s)	How intervention delivery may have been influenced
			• Lay counsellor on study leave during same 3-month period for retraining as pharmacy assistant	
	Individual health professional factors: knowledge and skills; cognitions; professional behaviour<br><br>*Professional interactions:* team processes<br><br>*Incentives and resources:* information system	• Routine data quality challenges	• Inconsistent application of data quality improvement activities – repeated training and supervision needed<br>• Communication between different cadres of staff (each responsible for completing different source documents) needed<br>• Poor documentation of tests performed and test results in source documents (patient medical records, clinic registers etc)<br>• Paper-based systems with multiple sources of information	• Missed opportunities for testing patients eligible for VL monitoring and repeat HIV testing<br>• Unreliable estimates of monthly testing targets for HIV VL or repeat HIV testing<br>• Unreliable estimates of progress towards monthly targets
		• HIV counselling and testing (general clinic) register not updated with patient's pregnancy status	• NGO assisting clinic with HIV testing (September 2016) was not documenting pregnancy status in HIV counselling and testing register	• Missed opportunities for testing patients eligible for repeat HIV testing<br>• Unreliable estimates of monthly testing targets for repeat HIV testing<br>• Unreliable estimates of progress towards monthly targets

*"Guidelines" referred to here are CQI tools and activities

CRH CQI team could only identify eligible patients for both VL and HIV re-testing based on clinic-based documentation (clinic registers) of HIV testing and positive diagnoses, ART initiation and VL monitoring as MCRs are retained by patients until delivery. Discrepancies between data sources were noted in all clinics at the start of CQI: registers vs tally sheets vs monthly summaries. This may have influenced targeted testing rates per month as well as interpretation of progress over time.

Observations described here were documented after the Intensive intervention phase had commenced (after the situational analysis) – some factors persisted after the situational analysis (eg, knowledge gaps in guidelines).

ANC, antenatal care; ART, antiretroviral therapy; CQI, Continuous Quality Improvement; CRH, Centre for Rural Health, University of KwaZulu-Natal (CQI mentors); eMTCT, elimination of mother-to-child transmission of HIV; MCR, maternity case record (antenatal medical record); MTCT, mother-to-child transmission of HIV; NGO, non-governmental organisation; PDSA, Plan-Do-Study-Act cycle.

Table S4. Summary of factors influencing delivery and 'normalisation' of the intervention reported by clinic healthcare providers (interview quotes): Tailored Implementation of Chronic Diseases (TICD) framework.

TICD framework category	Interview respondent*	Quote	How intervention delivery may have been influenced
Guidelines factors (CQI as a 'guideline'): feasibility of the recommended intervention; compatibility of the recommended behaviour with previous practice; effort; trialability; observability	Lay counsellor (**LC3**)	*"It's different right now, especially I will speak about the viral load for the pregnant female. It's different right now 'cause before we were not sure if we're missing some people… we were not taking bloods from them but now since we are recording, we can see the difference of how many people we were missing before since we were not recording."*	Improved evidence of better practice may have motivated staff to continue clinical documentation or comple registers<br>More time spent documenting may have been perceived as extra work
	Lay counsellor (**LC1**)	*"It was not easy. Because I'm working in counselling, I'm the only one counsellor….. on weekend I have to come and complete the tasks that I have to do."*	Difficult for staff on duty to find time to implement improvement activities
	Data capturer (**DC3**)	*"Yeah, but it's (CQI data variables in MONARCH) different from the government books. So, it's difficult to… there's some elements that MONARCH did…. wants us to work with. We don't have in the source documents, in the government source documents. So… sometimes it's difficult to work if …we don't have information."*	More time spent documenting perceived as extra work<br><br>Challenges maintaining extra source documents for patient tracking and tracing may threaten sustainability as not part of routine DoH monitoring & evaluation
	Data capturer (**DC1**)	*"There is a problem in that because when MONARCH team came, they want each team [member] per category [to be at meetings]…. Yes, others that are left, they want to know that what is happening."*	Non-clinic-CQI team unable to participate in CQI due to need to maintain clinical activities in parallel, were interested in CQI

TICD framework category	Interview respondent*	Quote	How intervention delivery may have been influenced
	Enrolled nurse (**EN1**)	*"Even yesterday they (CRH) came here and I wasn't there in that meeting because I was very busy helping on the ARTs, the sister on the ART side was not there so I have to go there."*	Difficult for staff on duty to find time to attend CQI meetings<br>Difficult for staff on duty to find time to implement improvement activities<br>Staff turnover likely to have slowed uptake of CQI skills and consistency of implementation
	Professional nurse (**PN1**)	*"It is effective..I know it is effective. I know because I see…"*	Improved motivation to continue implementing CQI due to visible improvements in patient care
Individual health professional factors: knowledge of own practice; skills needed to adhere; cognitions (including motivation, self-efficacy);	Data capturer (**DC3**)	*"It's (CQI) very interesting and it's very helpful, especially, it's like, it's an eye opening program. Like it helped us to understand also the guidelines."*	Improved motivation to take up and continue CQI due to novelty of intervention, supportive approach, relevance to current practice (guidelines implementation)
	Data capturer (**DC1**)	*"In our facility we have no operational manager...The one is acting and is busy like any other Sister. So we need someone who will told us what to do."*	Operational manager (leadership) presence required for decision making and ownership of improvement activities
	Professional nurse (**PN2**)	*"It was umm, it was interesting. We were able to see our shortfalls. We were able to, to see where we were doing wrong then, it helped us a lot."*	Improved motivation to take up and continue CQI due to novelty of intervention, supportive approach, relevance to current practice (guidelines implementation)<br>Non-judgmental approach to identifying gaps in clinical practice would have improved motivation
	Professional nurse (**PN3**)	*"...and they (CRH team) are so nice yes because they are trying to help us to improve our quality care to our patients, not that they are doing it for themselves they are doing it for us and for our patients. We appreciate although we are not doing well you see and we know."*	Improved motivation to take up and continue CQI due to novelty of intervention, supportive approach, relevance to current practice (guidelines implementation)<br>Non-judgmental approach to identifying gaps in clinical practice would have improved motivation

TICD framework category	Interview respondent*	Quote	How intervention delivery may have been influenced
	Professional nurse (**PN1**)	*"I don't want this MONARCH to leave us...MONARCH opens our eyes."*	Improved motivation to continue CQI due to supportive approach and relevance to current practice (guidelines implementation)<br>Need for ongoing support (unable to continue without external support or motivation: limited self-efficacy)
	Professional nurse (**PN3**)	*"..In terms of recording you see yeah our recording is very poor, I cannot write that because now you will see … our antenatal book that.. oh but we are trying.. maybe because there are many things we are doing as I am allocated here I am looking after the first ANC, the second ANC and … you see so it become very difficult."*	Poor documentation would have resulted in inaccurate measures of target testing rates at clinics<br>Measurement bias for primary endpoints in impact evaluation<br>Missed opportunities for testing, including risk of virologic failure or undiagnosed HIV
	Lay counsellor (**LC2**)	*"...to follow or to trace and track that woman... I was do as I'm doing my job. She's coming or not coming I was not give a damn but now I see the importance of why. I must make sure to get them and know where is she now..."*	Understanding rationale of guidelines is key to rigorous implementation<br>Different staff cadres working in silos, limited team work in general – lay counsellor not responsible for clinical decision making
	Lay counsellor (**LC1**)	*"It was not easy. Because I'm working in counselling, I'm the only one counsellor.…. on weekend I have to come and complete the tasks that I have to do."*	Difficult for staff on duty to find time to implement improvement activities
	Lay counsellor (**LC3**)	*"It is good because we have learnt something that we were not doing here at the clinic, like the recording. Now we know that we have to record everything that we are doing. Yes."*	Poor documentation would have resulted in inaccurate measures of target testing rates at clinics<br>Measurement bias for primary endpoints in impact evaluation<br>Missed opportunities for testing, including risk of virologic failure or undiagnosed HIV

TICD framework category	Interview respondent*	Quote	How intervention delivery may have been influenced
	Enrolled nurse (**EN2**)	*"I can say they do have skills. I can give them 60% on that...Maybe it's… they don't deal with those patients on daily basis as nurses. Honestly, the people who should be engaging themselves on this are the nurses, but then it's the people who are just doing minor, you see."*	Limited implementation of CQI due to limited participation in CQI by professional nurses<br>Lower cadre staff not empowered to transfer information up the hierarchy<br>Lower cadre staff not involved in clinical decision making
	Enrolled nurse (**EN2**)	*"You did a pledge; you can't say 'I solemnly pledge myself to the service of humanity' if you can't listen to other people when they say, 'ok, let's do this.'"*	Intrinsic motivation (medical ethics) to provide good quality care
	Enrolled nurse (**EN2**)	*"...because they were giving a chance to talk about what we have just understood and they gave us a chance to do the tasks that they were laid on the table. They were able to explain what QI is about. It was not like they had to explain everything and no exercises."*	Adult learning style – more interactive and interesting allows better engagement
	Operational manager (**OM1**)	*"..it is different because, I think we will have more healthy babies who are not infected with HIV because as I have said it was not easy for us to see those mothers, we would see them after their children became sick…"*	Intrinsic motivation (medical ethics) to provide good quality care<br>Understanding rationale of eMTCT guidelines
	Nutritional advisor (**NA2**)	*"..it was the way they introduced themselves, so it's fine. They didn't came here to change things, but they told us that they are here to help."*	Improved motivation to improve quality of care by feeling supported<br>Non-judgmental approach by external CQI providers
	Nutritional advisor (**NA1**)	(needing more training) *"Yes and viral load.... Others are taken viral load but not written down, yes."*	Persistent gaps in documentation may have resulted in measurement bias
Patient factors	Enrolled nurse (**EN2**)	*"..if there is a patient that is for, that is due for re-testing, you find that if you do a follow-up on*	Reduced successful tracking of patients for repeat HIV testing or VL monitoring

TICD framework category	Interview respondent*	Quote	How intervention delivery may have been influenced
		that patient, the cell phone isn't working. The cell phone that the patient left isn't working or is always off, and when you find… if you find the patient, the number that she gave wasn't hers. So you just have to leave a message; a message that you don't even know if it will be passed on."	Reduced motivation of staff to pursue the exercise of tracking patients if this is a frequent problem<br>Risk of undiagnosed HIV or VL failure
	Data capturer (**DC1**)	*"They (patients) are contactable although maybe for example; you give her return date of August then she decide to came in September. "*	Reduced motivation of staff to pursue the exercise of tracking patients if this is a frequent problem<br>Risk of undiagnosed HIV or VL failure<br>Measurement bias of targets
	Lay counsellor (**LC1**)	*"The rate for viral load now I think it's… because most of them they were starting clinic late, so now we have a understanding for early booking how to talk to them, yeah. And then how to manage those who's having a viral load, how to doing testing, it's easy for us now."*	Late ANC booking as a barrier to timely HIV testing and VL monitoring<br>Better understanding of how to manage patients (understanding of guidelines) facilitates better care
	Professional nurse (**PN4**)	*"They (patients) don't (forget)..when it's your day to come for results they come. If there is a problem they phone me.. all of them, they have my phone number."*	Motivated patients facilitate results follow-up and timely management
Professional interactions: communication and influence; team processes	Enrolled nurse (**EN2**)	*"I can say QI gave us team spirit. It made us be able to work together because… the only thing that brings people together is the group work....If you are an individual, you do not benefit, you just do what you are supposed to do but if you are working in groups you benefit a lot."*	Improved motivation to continue implementing CQI due to feelings of collegiality through group work
	Professional nurse (**PN2**)	*"Like MONARCH, MONARCH register, it ends up being used by a professional nurse and a data*	Improved motivation to continue implementing CQI due to feelings of collegiality through group work

TICD framework category	Interview respondent*	Quote	How intervention delivery may have been influenced
		capturer. Others are continuing with the other stuff and…we're… yes, we're quite a team."	
	Data capturer (**DC1**)	*"In our facility we have no operational manager...The one is acting and is busy like any other Sister. So we need someone who will told us what to do."*	Operational manager (leadership) presence required for decision making and ownership of improvement activities
Incentives and resources: availability of necessary resources; non-financial incentives and disincentives; continuing education system; assistance	Enrolled nurse (**EN1**)	*"Yeah is become difficult to get the QIT all of them at the same time when you have to have a meeting because the lay counsellor its only one, … no there are two but the other one is doing a training for pharmacy assistant …the lay counsellor is the one who have to do ongoing counselling, adherence, counselling, testing and everything so you only find that "ooohh today is a QIT meeting" but we try to attend the meeting."*	Difficult for staff on duty to find time to attend meetings or implement improvement activities
	Enrolled nurse (**EN1**)	*"...they must appoint new counsellors because people are there outside….if there is no counsellors in the clinic it won't work, it won't because as a nurse I have a job to do, vital signs, injections, what what..."*	Reluctance by other staff to take on additional duty of HIV counselling and testing due to increased workload<br>Absent lay counsellors during study leave or annual leave resulted in fewer repeat HIV tests being performed (noted by CRH in reports)
	Enrolled nurse (**EN2**)	*"The in-service training that we do as the clinic is far more different of the QI…because we just do it for the sake of doing it. Not that maybe it is important or we will benefit from it...While you on the other hand as MONARCH, you come up with something new. So something new is interesting."*	Improved motivation to learn and participate in the CQI intervention due to novelty

TICD framework category	Interview respondent*	Quote	How intervention delivery may have been influenced
	Operational manager (**OM1**)	*"I can say they (CRH team) are nice people, they are able to talk and you understand clearly what they are saying. But it did work but I was not always with them, to be clear."*	Improved motivation to continue implementing CQI due to feeling supported by the external CQI providers
	Lay counsellor (**LC1**)	*"...just to say thank you for giving us the support to our team. It helped us a lot."*	Improved motivation to continue implementing CQI due to feeling supported by the external CQI providers
	Lay counsellor (**LC1**)	*"I think now I can because on that time we're having a huge number of patient for testing that we are doing result testing. So it was hard for us, from now it's easy cause we having the.. an NGO that's helping us with testing…It's Humana."*	Improved motivation to continue implementing CQI due to feeling supported by NGO for HIV testing<br>Reduced motivation to participate in CQIdue to heavy workload
Capacity for organisational change: priority of necessary change	Operational manager (**OM1**)	*"You know people have a resistance to change but if you keep on emphasizing the importance of why are we doing this, they end up doing it."*	Emphasizing rationale for behavioural change may be a motivator
	Lay counsellor (**LC3**)	*"Before, it wasn't easy because they were… we were seeing like you are adding more job on top of what we were doing…But right now since we are getting used, it's getting easy. "*	Perceived extra workload would have resulted in resistance to engaging with CQI
	Professional nurse (**PN1**)	*"People will continue, except when… because there is a change over. You change to other clinics maybe, some people go to training, the new one comes I don't know… what will happen there but at the moment I think they will."*	Staff turnover will reduce available expertise and may demotivate ongoing implementation of CQI activities
	Enrolled nurse (**EN2**)	*"First few months will be good, we will do it. Maybe after three months, I don't think we will be doing it, not unless you will come and check…. We need a push. "*	Need for external motivation to continue with CQI

TICD framework category	Interview respondent*	Quote	How intervention delivery may have been influenced
	Data capturer (**DC1**)	*"In our facility we have no operational manager...The one is acting and is busy like any other Sister. So we need someone who will told us what to do."*	Operational manager (leadership) presence required for decision making and ownership of improvement activities

*Clinic details are not provided in order to maintain anonymity of respondents, given the small number of facilities and small number representing each staff cadre. Staff numbers are not in order of clinic randomisation

ANC, antenatal care; CQI, Continuous Quality Improvement; CRH, Centre for Rural Health, University of KwaZulu-Natal (CQI mentors); DC, data capturer; eMTCT, elimination of mother-to-child transmission of HIV; NGO, non-governmental organisation.
